# Prenylated Flavonoids of the Moraceae Family: A Comprehensive Review of Their Biological Activities

**DOI:** 10.3390/plants13091211

**Published:** 2024-04-27

**Authors:** Jaime Morante-Carriel, Suzana Živković, Hugo Nájera, Susana Sellés-Marchart, Ascensión Martínez-Márquez, María José Martínez-Esteso, Anna Obrebska, Antonio Samper-Herrero, Roque Bru-Martínez

**Affiliations:** 1 Plant Proteomics and Functional Genomics Group, Department of Biochemistry and Molecular Biology and Soil and Agricultural Chemistry, Faculty of Science, University of Alicante, Carretera San Vicente del Raspeig s/n, 03690 San Vicente del Raspeig, Alicante, Spain; hnajera@cua.uam.mx (H.N.); mjose.martinez@ua.es (M.J.M.-E.); anna.obrebska@ua.es (A.O.); antonio.samper@ua.es (A.S.-H.); roque.bru@ua.es (R.B.-M.); 2 Plant Biotechnology Group, Faculty of Forestry and Agricultural Sciences, Quevedo State Technical University, Av. Quito km. 1 1/2 vía a Santo Domingo de los Tsachilas, Quevedo 120501, Ecuador; 3 Institute for Biological Research “Siniša Stanković”—National Institute of Republic of Serbia, University of Belgrade, Bulevar Despota Stefana 142, 11108 Belgrade, Serbia; suzy@ibiss.bg.ac.rs; 4 Departamento de Ciencias Naturales, Universidad Autónoma Metropolitana–Cuajimalpa, Av. Vasco de Quiroga 4871, Colonia Santa Fe Cuajimalpa, Alcaldía Cuajimalpa de Morelos, Mexico City 05348, Mexico; 5 Research Technical Facility, Proteomics and Genomics Division, University of Alicante, 03690 San Vicente del Raspeig, Alicante, Spain; susana.selles@ua.es; 6 Multidisciplinary Institute for the Study of the Environment (IMEM), University of Alicante, 03690 San Vicente del Raspeig, Alicante, Spain; 7 Alicante Institute for Health and Biomedical Research (ISABIAL), 03010 Alicante, Alicante, Spain

**Keywords:** flavonoids, prenylated compounds, Moraceae family, bioactivities

## Abstract

Prenylated flavonoids (PFs) are natural flavonoids with a prenylated side chain attached to the flavonoid skeleton. They have great potential for biological activities such as anti-diabetic, anti-cancer, antimicrobial, antioxidant, anti-inflammatory, enzyme inhibition, and anti-Alzheimer’s effects. Medicinal chemists have recently paid increasing attention to PFs, which have become vital for developing new therapeutic agents. PFs have quickly developed through isolation and semi- or full synthesis, proving their high value in medicinal chemistry research. This review comprehensively summarizes the research progress of PFs, including natural PFs from the Moraceae family and their pharmacological activities. This information provides a basis for the selective design and optimization of multifunctional PF derivatives to treat multifactorial diseases.

## 1. Introduction

Plants, being immobile organisms, respond to various conditions by modifying the gene expression pattern of the proteins that regulate the production of metabolites. These metabolites are crucial for the interactions between a plant and its surroundings. Notably, polyphenols, as a vital category of specialized metabolites, are key players in shaping the physiological functions during the plant’s life cycle [1]. Over 8000 polyphenols are found in plants, which make them one of the most studied groups of plant metabolites [2]. A broad spectrum of monomeric and polymeric polyphenols is produced via a shikimate/phenylpropanoid pathway, including prenylated flavonoids (PFs) [3,4].

The flavonoid skeleton of polyphenolic phytochemicals contains prenyl side chains, and they originate from the secondary metabolic pathways of plants [5,6,7]. PFs are low-molecular-weight polyphenolics characterized by the prenyl side chains in the flavonoid skeleton [6,7]. In the plant kingdom, PFs are synthesized by incorporating an active isoprenoid molecule called dimethylallyl diphosphate (DMAPP) into flavonoid skeletons, including flavone, dihydroflavone, isoflavone, flavane, and chalcone [8]. The biosynthesis process is regulated by prenyltransferases, which are enzymes that determine the region and stereospecificity of the prenyl group incorporated into the flavonoid skeleton. This process contributes to the natural structural diversity of PFs [9,10,11].

PFs are more lipophilic than their non-prenylated counterparts. This characteristic results in a strong attraction towards P-glycoprotein in biological membranes. As a consequence, PFs can act as powerful inhibitors of P-glycoprotein. This is significant as it enhances flavonoids’ biological activities and pharmacological effects [12]. PFs have various pharmacological properties and benefit health by interacting with cellular targets involved in signaling pathways [13]. To study and/or obtain PFs, it has become important to apply structural modification strategies and explore methods to obtain PFs with notable pharmacological activities. Research on PFs for drug discovery has attracted great attention from both researchers and pharmaceutical companies due to their promising pharmacological properties [14]. Moreover, the study of PFs has recently become a hot topic due to their promising and diverse bioactivities in multitarget tissues. There are more than 800 species of monoecious and dioecious trees, shrubs, hemiepiphytes, climbers, and creepers belonging to the family Moraceae, commonly known as mulberries or figs, in the tropics and subtropics worldwide [15]. The aim of this review is to provide up-to-date information on the biological activities of PFs isolated from plants belonging to the Moraceae family.

## 2. Biosynthetic Pathway of Prenylated Flavonoids

Plant defense mechanisms rely heavily on the production of specialized metabolites known as phytoalexins, or PFs for short. These compounds are produced when the plant is under attack by pathogens or other stressors, and are synthesized from L-phenylalanine or L-tyrosine. The biosynthesis of PFs involves a series of intricate enzymatic reactions, each catalyzed by a specific enzyme: phenylalanine ammonia lyase (PAL), tyrosine ammonia lyase (TAL), cinnamate 4-hydroxylase (C4H), cytochrome P450 reductase (CPR), and 4-coumarate-CoA ligase (4CL). These enzymes, in a precise sequence, convert the starting amino acid into coumaroyl-CoA, which serves as the precursor for many different types of PFs [16]. Firstly, coumaroyl-CoA and three molecules of malonyl-CoA react with the help of the chalcone synthase (CHS) enzyme to form naringenin chalcone. This compound is then isomerized by the enzyme chalcone flavanone isomerase (CHI) to produce the basic skeleton of flavonoids. Finally, isoflavone is synthesized with the help of the 2-hydroxyisoflavanone synthase (IFS) and 2-hydroxyisoflavanone dehydratase (HID) enzymes [11,17]. Simultaneously, a lipophilic prenyl side chain is synthesized through the mevalonic acid (MVA) or methylerythritol phosphate (MEP) pathways [18,19].

**Figure 1:**
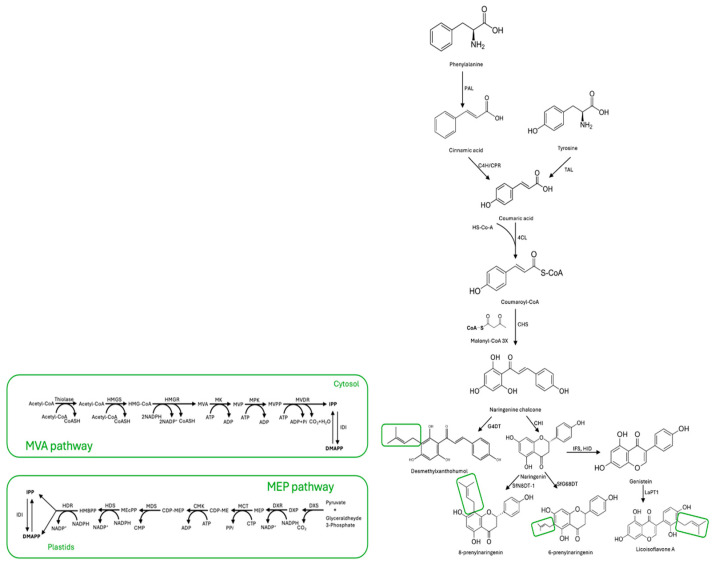
Metabolic pathway of prenylated flavonoids in plants. The small green box shows the prenylation sites related to MVA and MEP pathways. Abbreviations are as follows: MVA pathway: acetyl-CoA, acetoacetyl-CoA, thiolase; acetyl-CoA, HMG-CoA synthase (HMGS), 3-Hydroxy-3-methyl-glutaryl-CoA (HMG-CoA), HMG-CoA reductase (HMGR), mevalonic acid (MVA), mevalonic acid 5-kinase (MK); mevalonic acid phosphate (MVP), mevalonic acid phosphate 5-kinase (MPK); mevalonic acid 5-diphosphate (MVPP), mevalonic acid diphosphate decarboxylase (MVD); isopentenyl diphosphate (IPP), isopentenyl diphosphate isomerase (IDI), dimethylallyl diphosphate (DMAPP). MEP pathway: Isopentenyl diphosphate (IPP), Dimethylallyl diphosphate (DMAPP), Isopentenyl diphosphate isomerase (IDI), (E)-4-Hydroxy-3-methylbut-2-enyl diphosphate (HMBPP), (E)-4-Hydroxy-3-methylbut-2-enyl diphosphate reductase (HDR), 2C-methyl-D-erythritol 2,4-cyclodiphosphate (MEcPP), (E)-4-Hydroxy-3-methylbut-2-enyl diphosphate synthase (HDS), 4-(Cytidine 5′-diphospho)-2C-methyl-D-erythritol 2-phosphate (CDP-MEP), MEP-2,4-cyclodiphosphate synthase (MDS), 4-(Cytidine 5′-diphospho)-2C-methyl-D-erythritol (CDP-ME), Cytidyl MEP kinase (CMK), 2C-Methyl-D-erythritol 4-phosphate (MEP), MEP cytidyl transferase (MCT), 1-Deoxy-D-xylulose 5-phosphate (DXP), 1-Deoxy-D-xylulose 5-phosphate reductoisomerase (DXR), 1-Deoxy-D-xylulose 5-phosphate synthase (DXS), glyceraldehyde 3-phosphate + piruvate.

The MVA pathway is a biochemical process that begins with acetyl-CoA. In this pathway, three molecules of acetyl-CoA undergo reactions catalyzed by enzymes such as thiolase, HMG-CoA synthase, HMG-CoA reductase, mevalonic acid 5-kinase, mevalonic acid phosphate 5-kinase, mevalonic acid diphosphate decarboxylase, and isopentenyl diphosphate isomerase. These reactions ultimately produce two molecules, isopentenyl pyrophosphate (IPP) and its isomer DMAPP, used as plants’ primary prenyl donors [20]. The classic MEP pathway is a metabolic pathway in plants and bacteria that begins with the combination of pyruvate and glyceraldehyde phosphate, producing 1-Deoxy-D-xylulose-5-phosphate (DXP). DXP then goes through a series of transformations, resulting in the creation of other molecules such as 2-C-methyl-D-erythritol-4-phosphate (MEP), CDP-methyl-D-erythritol (CDP-ME), CDP-methyl-D-erythritol-2-phosphate (CDP-MEP), 2-methyl-D-erythritol-2,4-diphosphate (MEcPP), 1-hydroxy-2-methyl-2-butenyl-4-diphosphate (HMBPP), IPP, and DMAPP. This process involves seven enzymes: DXP synthase (DXS), DXP reductoisomerase (DXR), CDP-methyl-D-erythritol synthase (MCT), CDP-methyl-D-erythritol kinase (CMK), 2-C-methyl-D-erythritol-2,4-diphosphate (MDS), 1-hydroxy-2-methyl-2-butenyl-4-diphosphate (HDS), and 1-hydroxy-2-methyl-2-butenyl-4-diphosphate reductase (HDR) [19]. Subsequently, endogenous FPP synthase converts DMAPP and IPP into more extended prenyl donors, such as geranyl diphosphate and farnesyl diphosphate [10]. Furthermore, flavonoid prenyltransferase enzymes like SfFPT, SfN8DT-1, SfG6DT, and LaPT1 can produce diverse prenylated flavonoids by condensation of flavonoids with prenyl donors [8,11]. This is the most important step in the biosynthesis of PFs in plants. A scheme of the proposed metabolic pathway of PFs in plants is given in Figure 1.

## 3. Biological Activities of Prenylated Flavonoids

**Figure 2:**
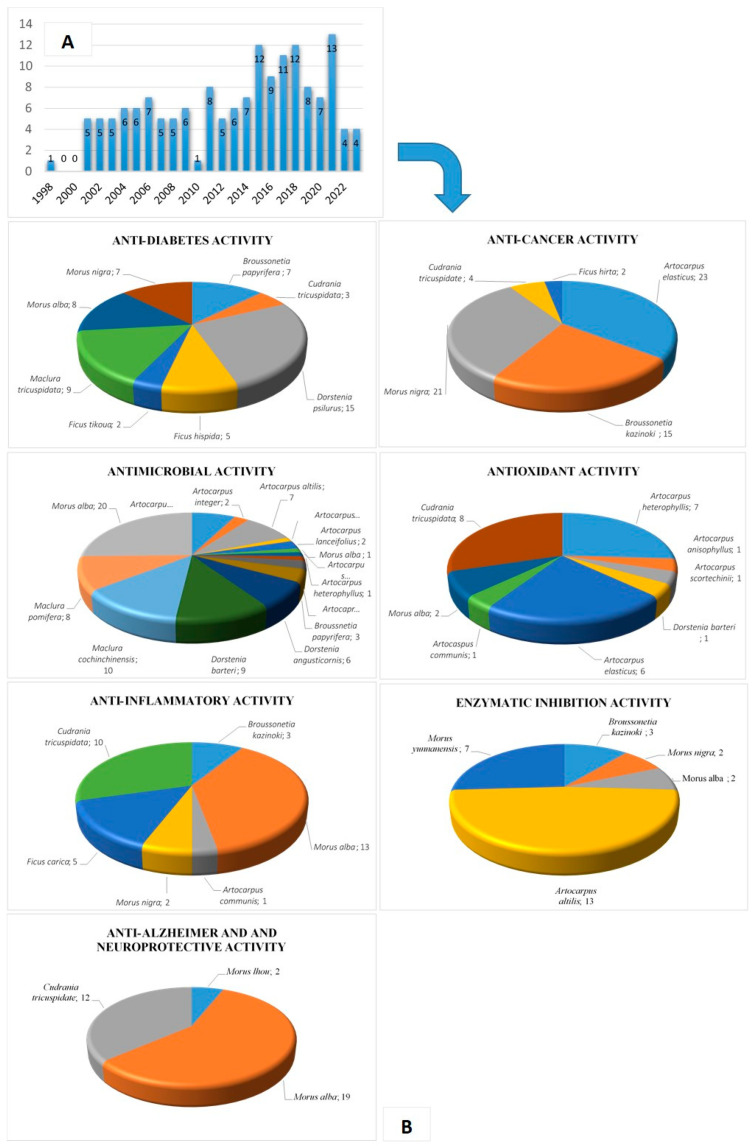
(**A**) Number of publications related to the PF compounds of the Moraceae family, according to PubMed and Web of Knowledge (1998–2023). (**B**) Number of PF compounds per bioactivity and species of the Moraceae family.

There is emerging evidence that PFs could protect plants from diseases by means of their strong inhibition of bacterial and fungal activities [10,11,21,22]. In addition, it has been suggested that prenylation increases the flavonoids’ lipophilicity, leading to increased affinity for biological membranes and improved interaction with target proteins [23]. This review mainly describes PFs’ biological activities, including anti-diabetic, anti-cancer, anti-inflammatory, antimicrobial, antioxidant, enzyme inhibitory, and anti-Alzheimer activities. We conducted a systematic literature search limited to 25 years (1998–2023) in the English language in PubMed. The search terms were “Moraceae prenylated flavonoids”, “Moraceae prenyl flavonoids”, “Moraceae prenylation flavonoids”, and the related activity combinations of these keywords via several search engines. In addition, Web of Knowledge was used as an alternative for the complete search of the relevant references. The articles extracted from these two databases revealed that most of the studies focused on the Moraceae family (Figure 2). Any articles with irrelevant research directions and little evidence were excluded.

### 3.1. Anti-Diabetic Activity

Growing interest is being shown in using natural products as remedies, especially for preventing and treating medical disorders. One of the therapeutic strategies to control hyperglycemia associated with type 2 diabetes is the targeting of enzymes *α*-amylase and *α*-glucosidase with not only novel inhibitors, but also with improved efficacy and minimal side effects [24]. Ryu et al. (2010) have reported that some PFs isolated from *Broussonetia papyrifera* roots exhibited significant *α*-glucosidase inhibitory activity. Of the tested compounds, flavanols papyriflavonol A and 8-(1,1-dimethylallyl)-5′-(3-methyl-but-2-enyl)-3′,4′,5,7-tetrahydroxyflavonol were the most potent *α*-glucosidase inhibitors, with IC_50_ values of 2.1 ± 0.2 μM and 3.6 ± 0.4 μM, respectively. Prenylated chalcone-broussochalcone A was also a very potent inhibitor, with an IC_50_ of 5.3 ± 0.3 μM, while broussochalcone B had an inhibitory potency of 11.1 ± 0.5 μM. Among flavans, diprenyl derivatives kazinol E and kazinol A showed similar inhibitory potency with 10.6 ± 1.5 μM and 12.0 ± 0.8 μM, respectively. In contrast, 3′-(3-methylbut-2-enyl)-3′,4′,7-trihydroxyflavane, with one prenyl group, displayed very low activity (IC_50_ of 75.7 ± 2.0 μM). From this, the authors concluded that an increased inhibitory potential correlates with the increasing number of prenyl groups in the tested compounds [25].

Protein tyrosine phosphatase 1B (PTP1B) negatively regulates the insulin signaling pathway and is, therefore, a promising target to treat various metabolic syndromes, including type 2 diabetes. Quang et al. (2015) evaluated the inhibitory effect of PFs isolated from *Cudrania tricuspidata* roots on PTP1B enzyme activity. They concluded that prenylated xanthones were more potent inhibitors compared to PFs, with IC_50_ values ranging from 1.9 ± 0.4 µM (for cudracuspixanthone A) to 4.6 ± 0.8 µM (for cudraxanthone L). One flavonoid, cudraflavanone D, showed the greatest PTP1B inhibition with an IC_50_ of 5.7 ± 1.5 µM. Based on an enzyme kinetic study, these authors also indicated the potential use of cudratricusxanthone N and cudraflavanone D to develop anti-diabetic drugs [26].

Tabopda et al. (2008) investigated the glycosidase inhibitory activity of prenylated flavonols dorsilurin F-K isolated from *Dorstenia psilurus* roots. The results showed that dorsilurin K (5,6-7,8-bis(2,2-imethyldihydropyrano)-3′-hydroxy-4′-prenyl-flavonol), with one prenyl group, had a very low inhibitory potential against *α*-glucosidase (IC_50_ of 43.95 ± 0.46 μM), while dorsilurin F (6,8,4′-triprenyl-5,7,3′-trihydroxyflavonol), with three unmodified prenyl groups, was the most potent *α*-glucosidase inhibitor with an IC_50_ value of 4.13 ± 0.12 μM [27]. However, despite dorsilurin F being more potent than the other tested compounds, it presented poor inhibitory activity against *β*-glucosidase and *α*-mannosidase, with IC_50_ values of 117.33 ± 0.15 and 192.09 ± 0.63 µM. Shi et al. (2016) found that the iso-PFs isolated from the twigs of *Ficus hispida,* isoderrone, 3′-(3-methylbut-2-en-1-yl) biochanin A, myrsininone A, ficusin A, and 4′,5,7-trihydroxy-6-[(1R*,6R*)-3-methyl-6-(1-methylethenyl)cyclohex-2-en-1-yl]isoflavone inhibited *α*-glucosidase activity with varying efficiency, and their IC_50_ values ranged from 22.1 ± 7.6 µM (for 3′-(3-methylbut-2-en-1-yl)biochanin A) to 108.1 ± 10.8 µM (for isoderrone) [28]. Similar results were reported by Fu et al. (2018) for 6-[(1R*,6R*)-3-methyl-6-(1-methylethenyl)-2-cyclohexen-1-yl]-5,7,4′-trihydroxyisoflavone and ficusin A isolated from *Ficus tikoua* rhizomes. These authors confirmed that both tested PF compounds had a moderate inhibitory effect against *α*-glucosidase, with IC_50_ values of 32.5 ± 6.7 µM and 84.6 ± 7.8 µM, respectively [29]. 

According to Jo et al. (2022), the prenylated isoflavonoids isolated from *Maclura tricuspidata* leaves had a considerable anti-diabetic potential and could be used to improve diabetic patients’ health. The vast majority of the tested PFs had a strong inhibitory effect on *α*-glucosidase, with IC_50_ values ranging from 3.2 µM (for millewanin G) to 23.5 µM (for erythrinin C). In addition, cudracusisoflavone M, euchrenone b_10_, 5,7-dihydroxy-6-(2″-hydroxy-3″-methylbut-3″-enyl)-4′-methoxylisoflavone, gancaonin M, alpinumisoflavone, 6,8-diprenylgenistein, isoerysenegalensein E, and senegalensin inhibited the formation of advanced glycation end products (AGEs) more efficiently than amino-guanidine (AG), which has been used as a positive control. On the PFs’ structure–activity relationship (SAR), these authors emphasized that the presence of hydroxyl groups in the isoflavonoid skeleton improved the inhibitory activity of PF compounds, while adding an OCH_3_ group caused the opposite effect [30]. 

Prenylated compounds chalcomoracin, moracin N, moracin C, and (2R)/(2S)-euchrenone a_7_, isolated from *Morus alba* leaves, displayed marked inhibitory activity against *α*-glucosidase, with IC_50_ values of 2.59 ± 0.24 µM, 2.76 ± 0.35 µM, 4.04 ± 0.84 µM, and 6.28 ± 1.01 µM, respectively [31].

Morusin isolated from *M. alba* root bark exhibited strong *α*-glucosidase inhibition, with an IC_50_ of 3.19 ± 2.10 μM, while dioxycudraflavone A showed a significantly lower inhibitory potential, with an IC_50_ value of 25.27 ± 3.11 µM [32]. 

Ha et al. (2018) investigated the anti-diabetic potential of iso-PF albanin T isolated from *M. alba* root bark. They concluded that this compound had more marked *α*-glucosidase inhibitory activity than that of the positive control acarbose, with an IC_50_ value of 35.35 ± 0.42 µM. In contrast, the inhibitory effect of albanin T on PTP1B was weak (IC_50_ > 100 µM) [33].

Zhao et al. (2018) used high-resolution *α*-glucosidase/*α*-amylase/PTP1B/radical scavenging profiling of PFs isolated from *M. alba* root bark. They demonstrated that iso-PFs were potent *α*-glucosidase inhibitors, with IC_50_ values ranging from 10.53 ± 1.10 µM to 38.81 ± 10.39 µM for kuwanon T and cyclomorusin, respectively [34]. Moreover, these two compounds together with kuwanon F strongly inhibited PTP1B, with the corresponding IC_50_ values coming close to or dropping below those of the control compound. The results also revealed that all tested PFs exhibited little inhibitory activity against *α*-amilase, with IC_50_ values ranging from 17.32 ± 18.97 to 69.38 ± 8.58 µM. 

In general, about 70 prenylated metabolites detected in *Morus* plants showed PTP1B inhibitory activity [35]. PF morunigrol B and morunigrol C, isolated from *Morus nigra* twigs, strongly inhibited PTP1B (over 90% inhibition when applied at 20 μg mL^−1^), with IC_50_ values of 7.7 ± 0.9 µM and 5.3 ± 1.8 µM, respectively. Conversely, morunigrol A, cudraflavone B, and morusin had a moderate inhibitory effect, with IC_50_ values ranging from 12.5 ± 1.3 µM to 22.1 ± 2.9 µM. As specialized cells of adipose tissue that store energy in the form of triglyceride droplets, adipocytes have been identified as a potential target for obesity and type 2 diabetes. Differentiated mature adipocytes exhibit specific markers, such as the gene expression of fatty acid-binding protein (aP2) and glucose transporter 4 (GLUT4), which are related to glucose transport and insulin sensitivity in adipose tissue and skeletal muscle [36]. Hu et al. (2011) investigated the effect of PFs isolated from *M. nigra* twigs on aP2 and GLUT4 gene expression in mouse fibroblast cells. The results showed that iso-PFs nigrasin H and nigrasin I increased triglyceride content (at a concentration of 50 µM and 25 µM, respectively, by 2- to 3-fold compared to the control), and the accumulation of intracellular lipid droplets. These compounds also stimulated adipocyte differentiation and induced the up-regulation of the gene expression and protein levels of aP2 and GLUT4 when applied at the 10 μM concentration [37]. 

Finally, several PFs (dorsilurin C and dorsilurin F-K) have been isolated from *Dorstenia psilurus* roots, and their α-glucosidase inhibitory activity has been evaluated [27]. Of them, dorsilurin F, with three unmodified prenyl groups, exhibited the best α-glucosidase inhibitory activity (IC_50_ 4.13 μM), while the dorsilurin K compound, with only one prenyl group, had the worst α-glucosidase inhibitory activity (IC_50_ 43.95 μM). The anti-diabetic activities of PFs from the Moraceae family are summarized in Table 1. 

### 3.2. Anti-Cancer Activity

Cancer is a major health threat and a significant challenge for humanity. It involves the abnormal growth of cells, which can lead to tumor formation. To reduce the risk of death from cancer or to stop its development, various anti-cancer drugs have been developed to treat different types of cancer, but they are often toxic to healthy cells as well as tumor cells. As a result, medicinal chemists are increasingly turning to natural products as a potential source of anti-tumor drugs. One such group of natural compounds are PFs, characterized by a prenyl side chain on the flavonoid skeleton. The prenyl side chain enhances the binding affinity of flavonoids to P-glycoprotein, resulting in a significant improvement in their biological activity [38]. Recently, PFs have been identified as compounds with possible cytotoxicity versus different cancer cells [39,40,41,42]. A screening test on human cancer cell lines is usually performed as a first step in the evaluation of chemicals for cancer prevention and treatment [20].

Several PF compounds (artelastin, artelastochromene, artelasticin, artocarpesin, cyclocommunin, artelastocarpin, and carpelastofuran) were extracted from *Artocarpus elasticus* and tested in vitro for their cytotoxicity against several human cell lines [20]. The results showed that the cytotoxic effect varied from strong to moderate. Artelastin, with three prenyl side chain substitutions of the A and C rings at positions 3, 6, and 8, respectively, demonstrated the most potent activity against three human cell lines, MCF-7, TK-10, and UACC-62, with an IC_50_ < 5 μM. This effect was particularly significant compared to the positive control doxorubin, which had IC_50_ values of 5.5, 57.0, and 9.4 μM, respectively [43,44]. Likewise, diprenylated flavonol broussonols A-E were isolated from the leaves of *Broussonetia kazinoki*, and their cytotoxicity against different cell lines was measured using an MTT assay. The results showed that broussonol D had the strongest cytotoxic activity against human KB tumor cells, with an IC_50_ value of 4.15 μM [45]. Further, some flavonol and hydroflavonol derivatives (cyclocommunol, artonin E, and broussoflavonol F) were obtained from *Artocarpus elasticus*. Among these compounds, artoindonesianin and artonin E, with a double bond between C-2 and C-3, and a prenyl sidechain at the C-3 position, were reported to have high cytotoxic activity against the murine leukemia P388 cell line, with IC_50_ values of 0.2 and 0.06 μg/mL. The results indicated that the prenyl sidechain at the C-3 position of prenylated flavones would elevate their antitumor activity [46,47].

Five PFs isolated from *Artocarpus heterophyllus*, 2-(4-hydroxy-phenyl)-8-(3-methyl-but-2-enyl)-chroman-4-one, bracteflavone B, dinklagin C, 6-(3-methyl-(E)-1-butenyl) chrysin, and 5,7,3′,5′-tetramethoxy-6-C-prenylflavone, showed potent antiproliferative activity in five human cancer cell lines (MCF-7, A-549, SMMC-7721, SW480, and HL-60 cells), with IC_50_ values ranging from 1.03 ± 0.06 µM to 22.09 ± 0.16 μM. These inhibitory effects were comparable to those of cisplatin or even more pronounced against most of the human cancer cell lines tested [48]. The prenylated flavones artocarnin A and carpachromenol as well as PFs cycloheterophyllin, artocarmin B, artocarmin C, gemichalcone B, artocarpusin A, gemichalcone A, gemichalcone C, isogemichalcone B, eleocharin A, 5,4′-dihydroxy-3′-methoxy-(6:7)-2,2-dimethylpyranoflavone, 2,4,2′,4′-tetrahydroxy-3-(3-methyl-2-butenyl)-chalcone, 6-prenyl-4′,5,7-trihydroxyflavone, and artocarpesin, isolated from the twigs of *Artocarpus nigrifolius*, showed in vitro cytotoxicity against the human cancer cell lines SiHa and SGC-7901. Among the PFs tested, eleocharin A was the most potent compound, showing high inhibitory activity against human gastric cancer cells SGC-7901 (IC_50_ of 8.3 ± 0.2 μM) and a significant cytotoxic effect on human cervical cancer cells SiHa (with an IC_50_ value of 0.7 ± 0.1 μM). At the same time, the inhibitory effect of two structurally similar analogues, 5,4′-dihydroxy-3′-methoxy-(6:7)-2,2-dimethylpyranoflavone and carpachromenol, significantly decreased against both cell lines. Accordingly, the authors speculated that the 3′-hydroxyl group might be crucial for the antiproliferative activity of eleocharin A. Gemichalcone B showed moderate inhibitory activity against SiHa cells (IC_50_ of 8.7 ± 0.2 μM), while isogemichalcone B and 5,4′-dihydroxy-3′-methoxy-(6:7)-2,2-dimethylpyranoflavone were weak inhibitors of SiHa cells, with IC_50_ values between 10 and 20 μM. Gemichalcone A showed low activity against SGC-7901 cells (IC_50_ 11.9 ± 0.1 μM). On the other hand, the compound 6-prenyl-4′,5,7-trihydroxyflavone moderately inhibited SGC-7901 cells (IC_50_ 9.6 ± 0.9 μM) but showed weak activity against SiHa cells (IC_50_ 13.3 ± 0.4 μM). The other PF compounds isolated from *A. nigrifolius* showed low cytotoxicity against both tested human cancer cell lines in vitro, with IC_50_ values above 20 μM [49].

Artonin E from the bark of *A. elasticus* showed strong cytotoxic activity against human estrogen receptor (ER+)-positive (MCF-7) and human estrogen receptor (ER−)-negative breast cancer cells (MDA-MB 231), with IC_50_ values of 2.6 ± 0.3 μg mL^−1^ and 13.5 ± 1.3 μg mL^−1^, respectively. In contrast, this compound showed rather weak inhibition against the human hepatocarcinoma cell line (HepG2), with an IC_50_ of 33.8 ± 1.1 μg mL^−1^, suggesting that the cells might have a different specific molecular site for ligand–receptor interactions [50]. 

Eleven prenylated isoflavone derivatives isolated from the fruits of *Ficus altissima* were evaluated for their antiproliferative activities against a human liver tumor cell line (HepG2) and two types of human breast tumor cell lines (MCF-7 and MDA-MB-231). Ficusaltin B, lupinalbin D, and isolupalbigenin showed potent antiproliferative activities against human liver cancer cells HepG2, with IC_50_ values of 37.50 ± 3.66 µM, 37.52 ± 2.77 µM, and 40.00 ± 2.37 µM, respectively. In addition, ficusaltins A and B together with lupinalbin D showed a significant inhibition of proliferation of high-grade metastatic human breast cancer cells MDA-MB-231, with IC_50_ values ranging from 15.44 ± 0.69 μM to 39.79 ± 2.72 μM. Ficusaltin B displayed the strongest proliferation inhibitory effect against four tested cell lines, with the highest selectivity index (SI = 2.78) for MDA-MB-231 cells. On the other hand, lupinalbin D showed relatively high selectivity for HepG2 and MDA-MB-231 cell lines, with SI values of 1.90 and 2.02, respectively. The other PF compounds tested showed weak antiproliferative activities against the tumor cells tested (with IC_50_ values above 100 μM). It was hypothesized that the presence of a prenyl group at C-6 or C-8 in the A ring together with a prenyl group at C-3′ in the B ring might be essential for the antiproliferative effects of the tested prenylated isoflavones [51].

Zelova et al. (2014) investigated the cytotoxicity of the PFs kuwanon E, kuwanon C, sanggenon H, cudraflavone B, and morusinol, isolated from the root bark of *M. alba*. Kuwanon C was found to have the greatest inhibitory effect against the human monocytic leukemic cell line THP-1, with an IC_50_ of 1.7 ± 0.03 μM. It was hypothesized that the high toxicity of kuwanon C compared to kuwanon E (IC_50_ of 4.0 ± 0.08 μM) was due to the presence of two prenyl groups in kuwanon C and the different position of the prenyl group of kuwanon E. Furthermore, the authors concluded that the differences between the activities of morusinol (IC_50_ of 4.3 ± 0.09 μM) and sanggenon H (IC_50_ > 10 μM) were due to the double prenyl substitution of rings A and C in morusinol compared to a single cyclic prenyl group on ring B in sanggenon H. Sanggenon E, extracted from the root bark of *M. nigra*, also showed high cytotoxicity against THP-1 human monocytic leukemic cells, with an IC_50_ value of 4.0 ± 0.12 μM [52].

According to Li et al. (2018), the diprenylated flavone dioxycudraflavone A, isolated from the *M. alba* root bark, showed a moderate antiproliferative effect against NCI-H292 and A549 lung cancer cells, with IC_50_ values of 17.80 ± 0.57 μM and 29.2 ± 1.79 μM, respectively [32]. At the same time, morusin showed significant cytotoxicity against the tested tumor cells (IC_50_ ~ 9 μM). A comprehensive review of the antitumor properties of morusin, with particular emphasis on in vitro and in vivo studies, was recently published by Hafeez et al. (2023) [53].

Among 25 different PFs isolated from the root bark of *M. alba*, only benzokuwanon E significantly inhibited the viability of gastric cancer cells HGC27 in vitro (46.84 ± 3.02%) and showed some cytotoxicity, with an IC_50_ value of 10.24 ± 0.89 μM [54].

Su et al. (2023) combined kuwanon A from the root bark of *M. alba* with 5-fluorouracil to reduce the progression of gastric cancer (GC). Kuwanon A was found to significantly inhibit the growth of all tested human GC cell lines, including HGC-27, BGC-823, MKN-45, and SGC-7901, with IC_50_ values of 13.84 μM, 18.27 μM, 21.28 μM, and 39.32 μM, respectively. The authors also demonstrated that kuwanon A induced cell cycle arrest and apoptosis in the GC cells tested [55].

Nine PFs isolated from the leaves of *Morus alba* (3′-geranyl-3-prenyl-2′,4′,5,7-tetrahydroxyflavone, 3′,8-diprenyl-4′,5,7-trihydroxyflavone, kuwanon S, sanggenon J, sanggenon K, cyclomulberrin, cyclomorusin, morusin, and atalantoflavone) showed significant cytotoxic activity against human cervical carcinoma cells HeLa, human breast carcinoma cells MCF-7, and human hepatocarcinoma cells Hep3B compared to antitumor agent deguelin, used as a control compound [56]. Of the compounds tested, morusin showed the strongest cytotoxicity against HeLa cells, with an IC_50_ value of 0.64 ± 0.14 μM, while the cytotoxicity of the other PF ranged from 1.64 ± 0.21 μM to 3.69 ± 0.86 μM detected for kuwanon S and cyclomulberrin, respectively. On the other hand, sanggenon K was most effective against the MCF-7 and Hep3B cell lines (with IC_50_ values of 3.21 ± 0.87 μM and 3.09 ± 0.67 μM, respectively). A comparison of the compounds structures and their cytotoxicity suggested that 3′-geranyl-3-prenyl-2′,4′,5,7-tetrahydroxyflavone and morusin with an additional prenyl side chain at the C-3 position exhibited stronger cytotoxicity than kuwanon S and atalantoflavone. This implied that the prenyl side chain at the C-3 position might contribute to the cytotoxicity of PFs against human cancer cells. The anti-cancer bioactivity of PF compounds of the Moraceae family are summarized in Table 1.

### 3.3. Antimicrobial Activity

Four new prenylated dihydrochalcones, named elastichalcones C-F, were isolated together with elastichalcone B from *Artocarpus elasticus* leaves and subsequently tested for their antibacterial activity against *Staphylococcus aureus* (SA) and methicillin-resistant *S. aureus* (MRSA) strains [57]. Elastichalcone C inhibited SA and MRSA growth, with MIC values of 19.5 and 9.75 µM, respectively. The other PFs showed rather weak activity. In addition, elastichalcones B and C possessed the highest bactericidal potential against SA of all tested PFs (with an MBC value of 156.02 µM), while elastichalcone F was the most effective against MRSA, with an MBC value of 174.79 µM.

Artocarpin and cudraflavone C, which were isolated from *Artocarpus integer* root extract, also showed antibacterial activity against *S. aureus*, *S. epidermidis* and *Propionibacterium acnes*, an anaerobic pathogen that causes acne vulgaris or acne inflammation, with MIC values of 2, 4, and 2 µg mL^−1^, respectively [58]. In addition, cudraflavone C from *Artocarpus hirsutus* Lam. stem bark had remarkable antibacterial properties against a variety of multidrug-resistant *S. aureus* strains, including MRSA, VRSA, and VRE, with an MIC value of 4 μg mL^−1^. This prenyl flavone also had a synergistic bactericidal effect when used in combination with gentamycin, as well as a marked anti-biofilm property compared to levofloxacin and vancomycin [59]. Similarly, artocarpin, isolated from *A. hirsutus* Lam stem bark, and subsequently tested against multiple pathogens like various *S. aureus* and *Enterococcus* sp. strains, had strong antibacterial and anti-biofilm effects without inducing resistance, regardless of repeated treatments [60].

A prenylated flavone compound, 14-hydroxyartonin E, isolated from *Artocarpus lanceifolius* Roxb bark, exerted moderate activity against Gram-positive bacteria *S. aureus* and *Streptococcus pneumonia*, and had a low antimicrobial potential with Gram-negative bacteria *Salmonella thyposa* and *Escherichia coli* [61].

Of the other bioactive compounds, *Artocarpus champeden* is a rich source of PFs. Hu et al. (2023) isolated 11 PFs from *A. champeden* twigs and subsequently determined their activities against plant pathogenic fungi, *Golovinomyces cichoracearum* (DC.), which cause powdery mildew disease in tobacco and other plant species. The results revealed that one of the novel PFs, named compound 1 (4′-hydroxy-8-methoxy-6-(4-methyl-1H-pyrrol-2-yl)-flavone), exhibited greater antifungal activity than the control compound carbendazim, with an IC_50_ value of 51.5 μg mL^−1^ and a corresponding inhibition rate of 88.3% ± 6.2. The compound identified as 4′-hydroxy-8-methoxy-6-(4-methylfuran-2-yl)-flavone, together with 2″,2″-dimethylpyran-(5″,6″:6,7)-5,4′-dihydroxy-4′-methoxy-flavonol and corylifol C, showed high inhibition rates above 65% [62].

Boonphong et al. (2007) isolated prenylated flavones with antitubercular and antiplasmodial potentials from *Artocarpus altilis* roots. Of the tested compounds, artocarpin possessed the greatest activity against *Mycobacterium tuberculosis*, with an MIC of 3.12 μg mL^−1^, but significantly less antitubercular activity was observed for morusin, cudraflavone B, and cudraflavone C (MIC within the 12.5–50 μg mL^−1^ range). Regarding the quantitative evaluation of PFs’ activity against the multidrug-resistant strain of *Plasmodium falciparum*, the results showed a moderate antimalarial potential of all tested compounds, with MIC values falling within the 1.9–4.3 μg mL^−1^ range [63].

In a similar study by Widyawaruyanti et al. (2007) on the anti-malarial activity of PFs isolated from *A. champeden*, the PF compound heteroflavone C was the most potent inhibitor of *P. falciparum* activity, with a significantly lower IC_50_ value (1 nmol L^−1^) than that reported for the control compound chloroquine (with an estimated IC_50_ value of 6.3 nmol L^−1^). The activity of the other tested PFs varied within the range from 0.001 to 75.3 µmol L^−1^ [64].

Mustapha et al. (2010) investigated the effect of PFs from different *Artocarpus* species (*A. heterophyllus*, *A. elasticus*, and *A. lanceifolius*) against two *P. falciparum* strains, namely, K1 and 3D7 [65]. Artonin E inhibited both tested strains, with IC_50_ values of 0.1 µg mL^−1^ and 0.3 µg mL^−1^, respectively. However, the related compound of the 3-prenylflavone type, 12-hydroxyartonin E, had a stronger inhibitory effect against the K1 strain (IC_50_ 0.9 µg mL^−1^). Similarly, artonin E, isolated from *A. rigida*, showed marked antiplasmodial activity against the chloroquine-resistant strain of *P. falciparum* 3D7 in vitro, with an IC_50_ value of 0.2 μg mL^−1^ [66].

Govender et al. (2023) performed molecular docking and molecular dynamics simulations to screen some of the PFs found in *Artocarpus altilis* for their potential inhibitory activities against SARS-CoV family receptors. In addition, the most stable PF-bound SARS-CoV receptor complexes were validated with PFs as target ligands. Of all the evaluated combinations, 5RE4-artocarpin and 5RE4-artoindonesianin V exhibited the greatest hydrophobic interactions, with minimum binding affinities (MBAs) between −6.6 kcal mol^−1^ and −6.4 kcal mol^−1^. Accordingly, it was highlighted that the PFs artocarpin and artoindonesianin V might be good potential inhibitors for SARS-CoV family receptors [67].

The antimicrobial effect of prenylated flavonol papyriflavonol A and isoflavanone broussochalcone A, as well as the PF compound called kazinol B (obtained from *Broussnetia papyrifera* (L.) Vent. root bark), were tested with some fungi (*C. albicans* and *S. cerevisiae*), Gram-negative bacteria (*E. coli* and *S. typhimurium*) and Gram-positive bacterial strains (*S. epidermis* and *S. aureus*). After the analysis, the above-mentioned prenylated compounds were divided into subgroups based on their antimicrobial potential. Thus, papyriflavonol A showed strong antibacterial and antifungal activity (with the lowest MIC values of 10 and 12.5 µg mL^−1^, respectively), kazinol B displayed specific activity against Gram-positive bacteria (with an MIC of 20 µg mL^−1^), while broussochalcone A possessed moderate activity against *C. albicans*, with an MIC of 45 µg mL^−1^ [68].

A wide variety of bioactive prenylated compounds were identified in *Dorstenia* species, mainly belonging to mono-, di-, and triprenylated flavonoids [69]. Kuete et al. (2007) evaluated the antimicrobial activity of the four PFs found in *Dorstenia angusticornis* twigs: gancaonin Q, stipulin, angusticornin B, and bartericin A. The in vitro antimicrobial tests included 13 g-negative, 6 g-positive bacteria, and 3 *Candida* species (22 microbial strains in all). According to the results, the tested compounds had significant antibacterial and anticandidal activity. For example, angusticornin B and bartericin A inhibited the growth of all tested pathogens, while gancaonin Q and stipulin together were effective against *Candida* and 77.27% of the tested bacterial species. Similarly, the minimum inhibitory concentration (MIC) for PFs ranged from 0.31 to 78.12 µg mL^−1^ for angusticornin B, and from 0.61 to over 78.12 µg mL^−1^ for gancaonin Q and stipulin. At the same time, values below 0.31 µg mL^−1^ and up to 39.06 µg mL^−1^ were determined for bartericin A. It should be noted that the MIC values obtained for the tested compounds were equal to or lower than the reference antibiotics, which emphasized the great antimicrobial activity of the PFs isolated from *Dorstenia angusticornis*. In addition, the minimum microbicidal concentration (MMC) results also confirmed the strong antimicrobial activity and selectivity of all the PF compounds tested [70]. In the corresponding study by Mbaveng et al. (2008) on the antimicrobial activity of the PFs isolated from *Dorstenia barteri* twigs, it was demonstrated that isobavachalcone and kanzonol C were effective against the growth of all the microbial species tested [71]. Amentoflavone and 4-hydroxylonchocarpin had a selective inhibitory effect on 50% and 77.3% of the tested microorganisms, respectively. As for MIC, the lowest value of 0.3 µg mL^−1^ was obtained for isobavachalcone, which indicated by far the strongest antimicrobial potential of all tested PFs compared to the reference antibiotics (RAs). In addition, the MIC values of 1.2 µg mL^−1^ and 4.9 µg mL^−1^ obtained for 4-hydroxylonchocarpin and kanzonol C were also lower than or equal to those of RAs. For SAR, the authors speculated that the addition of the second 3-isoprenyl group to isobavachalcon, which led to kanzonol C, significantly decreased its antimicrobial activity. The cyclisation of isobavachalcon (to form 4-hydroxylonchocarpin) could also significantly reduce this compound’s activity. According to Song et al. (2021), isobavachalcone not only showed strong bactericidal activity against Gram-positive bacteria, with MIC values of 1–4 μg mL^−1^, but also recovered the susceptibility of colistin against some Gram-negative bacterial strains [72].

Dzoyem et al. (2013) evaluated the activity of four PFs from the *Dorstenia* species, namely, 6,8-diprenyleriodictyol, isobavachalcone, 6-prenylapigenin, and 4-hydroxylonchocarpin, against the clinical isolates of both methicillin-sensitive (MSSA) and methicillin-resistant *S. aureus* (MRSA) strains, as well as four pathogenic fungal species. This work also included a study about the mechanisms underlying the antibacterial activity of the selected PFs and assessed their toxic effects. The results revealed the low in vivo toxicity and remarkable bactericidal/bacteriolytic activity of 6-8-diprenyleriodictyol, followed by the potent antimicrobial effect of isobavachalcone and 4-hydroxylonchocarpin against the tested bacterial and fungal pathogens. These three PFs showed significant bactericidal activity against all *S. aureus* strains, with MIC values ranging from 0.5 to 16 μg mL^−1^. In addition, the effect of 6,8-diprenyleriodictyol on one of the MSSA strains of *S. aureus* was similar to that of both RA-gentamicin and norfloxacin (MIC of 0.5 μg mL^−1^). A strong antifungal effect of 4-hydroxylonchocarpin against one of the *Cryptococcus neoformans* strains was observed, with a similar MIC value to that of the control amphotericin B (MIC of 0.5 μg mL^−1^). In contrast, 6-prenylapigenin exhibited the lowest antimicrobial potency of all tested PFs [73].

Based on the phytochemical analysis of *Maclura cochinchinensis* Corner leaf and fruit extracts, four new isoflavones (macluracochinones A–D) and one flavone (macluracochinone E) appeared and were characterized, together with other previously known compounds [74]. The authors investigated the antimicrobial activities of 12 selected compounds using five Gram-positive, two Gram-negative bacteria, and one fungal species. According to the results, isoflavones gancaonin M, lupiwighteone, lupalbigenin, warangalone (scandenone), auriculatin, and millexatin F displayed remarkable activities against *C. albicans* and all tested Gram-positive bacteria, with MIC values falling within the range of 2–8 μg mL^−1^ and 1–8 μg mL^−1^. Lupalbigenin was particularly effective against *Staphylococcus* strains, including MRSA, with an MIC of 1 μg mL^−1^. The same MIC value also appeared for millexatin F against *S. aureus*. The other analyzed compounds, namely, alpinumisoflavone, derrone, 7-O-(2,2-dimethylallyl)-aromadendrin, cochinchinone A, xanthone V1, and a novel flavone, showed variable antimicrobial activity against different Gram-positive bacterial strains and *C. albicans*. The determined MIC values ranged from 4 to 128 μg mL^−1^ and from 32 to 128 μg mL^−1^. Interestingly, none of the tested PFs exhibited antimicrobial activity against Gram-negative bacteria *Shigella flexneri* and *Salmonella typhimurium*, with the exception of lupiwighteone, which displayed rather weak activity, with an MIC of 128 μg mL^−1^ [74].

The antimicrobial activity of warangalone (scandenone), isolated from *Maclura pomifera* (Rafin.) Schnider fruit, was also investigated by Özçelik et al. (2006) [75]. The authors noted that this PF was the most effective one against *S. aureus* and *E. faecalis*, with the same MIC value of 0.5 μg mL^−1^, followed by *E. coli* (MIC of 2 μg mL^−1^) and *K. pneumoniae* (MIC of 4 μg mL^−1^). The antibacterial activity of the tested compound against the other strains used in their study was weaker and ranged between 8 and 32 μg mL^−1^. Warangalone showed a significant antifungal potential against *C. albicans*, with similar MIC values to those of ketoconazole (1 μg mL^−1^) [75].

Species from the genus *Morus* are considered a very rich source of biologically active compounds, including a variety of PFs with a significantly high antimicrobial potential. Sanggenol A, sanggenon C, sanggenon D, and kuwanon E, extracted from mulberry (*M. alba*) root bark, inhibited *S. aureus* growth, with MIC values ranging from 17.6 μmol L^−1^ for sanggenon D to 70.6 μmol L^−1^ for sanggenon C. The antibacterial activity of the PFs tested against *E. coli* was much lower. The proposed mechanism of action of sanggenon D might involve changes in the fatty acid metabolic pathway, which would consequently affect the biosynthesis of the bacterial cell membrane [76].

According to Čulenová et al. (2020), six PFs isolated from *M. alba* root bark showed antibacterial activities against three MRSA, one *E. faecalis*, and three vancomycin resistant *Enterococcus* strains (VRE) [77]. Kuwanon C, kuwanon E, kuwanon T, and morusin were equally or more effective than ampicillin and ciprofloxacin, with MICs of 2–8 μg mL^−1^, while kuwanon U exhibited moderate activity against the tested MRSA strains. Additionally, kuwanon C, kuwanon T, and morusin strongly inhibited *E. faecalis* growth, with MICs of 4–8 μg mL^−1^, while kuwanon C and morusin exhibited stronger antimicrobial activity than vancomycin against all the VRE strains, with MIC values from 4–8 μg mL^−1^ [77]. Prenylated flavones kuwanon C, kuwanon T, and morusin generally possessed the highest anti-MRSA potential, with diprenylated compounds kuwanon C and T showing similar activity levels. However, morusin was significantly more potent than morusinol with the modified C-3 prenyl group. These results were consistent with the previous work of Wu et al. (2019), who reported the same MIC value for morusin against MRSA and the *E. faecalis* strains. However, the authors revealed that this compound was not effective against the tested Gram-negative bacteria, namely, *E. coli*, *P. aeruginosa*, and *K. pneumonia* [78].

Zhu et al. (2021) evaluated the anti-MRSA bioactivity of PFs isolated from the *M*. *alba* root bark. Kuwanon C, kuwanon B, kuwanon E, 5′-geranyl-5,7,2′, 4′-tetrahydroxy-flavone, and morusin possessed the strongest antibacterial activity of all tested PFs, with MIC and MBC values of 2–4 μg mL^−1^ and 2–8 μg mL^−1^, respectively, compared to kanamycin and ampicillin [79]. Kuwanon U showed better anti-MRSA bioactivity than berberine and ampicillin, with MIC values of 4–16 μg mL^−1^ and MBC values of 8–32 μg mL^−1^. 

As PFs have potent anti-MRSA activity, they can be a valuable source for developing new antibacterial therapies. Some recent studies have addressed the synergistic effect of PFs that derive from Moraceae species with conventional anti-infective remedies. For example, Zuo et al. (2018) showed the anti-MRSA synergism of four PFs from *Morus alba* root bark with 11 antibacterial agents against clinical MRSA isolates. Kuwanon E, cyclocommunol, and morusin were able to exhibit potent antibacterial activity against MSSA/MRSA strains, with MIC values within the range of 4–16 μg mL^−1^, 8–16 μg mL^−1^, and 8–32 μg mL^−1^, respectively [80]. Although morusinol displayed the weakest antibacterial activity of all tested PFs, its marked synergistic affect with amikacin and streptomycin were demonstrated against all 10 MRSA isolates. Similarly, kuwanon E, together with morusinol, had synergistic effects with etimicin, vancomycin, and ciprofloxacin against most of the isolates used. It could be generally concluded that compounds cyclocommunol and morusinol covered the broadest spectrum of synergistic effects with all antibacterial agents. Furthermore, the authors emphasized that MRSA resistance to aminoglycosides, especially amikacin, could be reversed by all the used PFs [80].

A similar study on the prenylated phenolics from *Morus alba* L. root bark involved investigating the antibacterial (antistaphylococca) potential of PFs both alone and in combination with commonly used antibiotics [81]. Six PFs (kuwanon C, kuwanon E, kuwanon T, kuwanon U, morusin, and morusinol) displayed strong antibacterial activity against MSSA/MRSA strains, even at lower doses than commonly used antibiotics, with MIC values between 2 and 8 μg mL^−1^. When substituted for free prenyl (morusin), diprenyl (kuwanon C and kuwanon T), or geranyl moieties (kuwanon E and kuwanon U), no significant change in activity occurred. The C3 modification of the prenyl moiety with a hydroxyl group (e.g., morusinol) reduced bactericidal activity (MBC ≥ 64 μg mL^−1^) compared to morusin. In addition, four of the tested PFs, namely, kuwanon C, kuwanon E, kuwanon T, and morusin, had a synergistic effect with kanamycin, with kuwanon C proving to be the most effective. In addition, kuwanon C, kuwanon T, and morusin in combination with oxacillin enhanced the effect of this cell wall-active antibiotic, while morusin alone showed a partial synergism in all the resistant MRSA strains to ciprofloxacin. According to Aelenei et al. (2020), morusin was able to reverse the oxacillin resistance of MRSA and could lower its MIC to 1 μg mL^−1^. Additionally, this PF showed a synergistic effect with various antibiotics against *S. epidermidis* by significantly reversing its resistance to tetracycline [82]. Table 1 summarizes the specific information of PF compounds of the Moraceae family with antimicrobial activity.

### 3.4. Antioxidant Activity

PFs are known to have the remarkable ability to scavenge free radicals with low IC_50_ values, including DPPH radicals, superoxide anion radicals, and hydroxyl radicals [83]. The high proportion of free hydroxyl groups in flavonoids may contribute to their antioxidant activity [34]. In addition, prenylation may significantly affect flavonoids’ antioxidant activity depending on the assay and prenylation pattern. For example, the flavones with prenyl groups (i.e., artonins A and B and cycloheterophyllin isolated from *Artocarpus heterophyllis)* were potent antioxidants, whereas other prenyl flavones (i.e., artocarpine, artocarpetin, artocarpetin A, and cycloheterophyllin diacetate and peracetate) were ineffective [84]. In addition, PFs generally showed lesser scavenging activity during in vitro DPPH radical assays compared to parent flavonoids [85].

Most PFs, with C-prenyl being commoner than O-prenyl derivatives, have been identified as chalcones, dihydrochalcones, flavones, flavanones, flavonols, and isoflavones. Prenyl side chains can involve variations in the number of carbons, dehydration, cyclization, oxidation, or reduction, which might result in a wide range of compounds with a remarkable antioxidant potential [38]. The commonest substitution in the flavonoids with antioxidant properties is the 3,3-dimethylallyl chain. l,l-dimethylallyl, geranyl, lavandulyl, and farnesyl units may also be present, and occur in chalcones, dihydrochalcones, flavones, flavanones, isoflavones, xanthone-like, and other complex molecules [86].

Chalcone derivatives are the most abundant class of PFs with significant antioxidant activity. However, only a limited number of these derivatives were detected in Moraceae plants, such as isobavachalcone from *Artocarpus anisophyllus* heartwood and leaves [87], flemichapparin A from *Artocarpus scortechinii* leaves and stem bark [88], or isobavachalcone and bartericin A from *Dorstenia barteri* twigs and leaves [89]. Furthermore, naturally occurring flavone derivatives were obtained mainly from the Moraceae plants belonging to *Artocarpus*, *Cudrania*, and *Dorstenia* species [86]. All natural prenyl flavones with antioxidant activity are C-substituted and mostly mono- and di-prenylated. The only proven triprenylated derivative, artelastoheterol, was isolated from *Artocarpus elasticus* [90]. Several flavanones, which are the second most abundant class of PFs with antioxidant activity, were also isolated from *Artocarpus*, *Dorstenia*, and *Cudrania* species. The prenylated isoflavones found in Moraceae plants are predominantly prenylated on ring A, such as 6-prenyl or 8-prenyl derivatives, as well as some derivatives with a 6,7-(2,2-dimethylchromene)-fused unit. Interestingly, all prenylated xanthone-like compounds were isolated from different *Artocarpus* species [86].

A variety of PFs found in *Artocapus* species were tested for their DPPH• scavenging activity. Artoflavone A, isolated from *A. communis* root bark [91], as well as flavone artelastoheterol and xanthone-like compounds cycloartobiloxanthone and cycloartelastoxanthone, isolated from *A. elasticus* root bark [90], showed concentration-dependent DPPH• scavenging activity, with IC_50_ values of 24.2 ± 0.8, 42.2 ± 2.8, 26.8 ± 1.2, and 18.7 ± 2.2 μM, respectively. The IC_50_ of BHT and α-tocopherol, used as positive controls, were 80.0 ± 10.9 and 18.1 ± 1.5 μM, respectively. Xanthone-like compounds cyclogeracommunin and artonol A, isolated from these species, were unable to scavenge DPPH• under the same experimental conditions. Based on the obtained results, the authors found that the 2,2-dimethylpyran ring substitution at C-7 and C-8 of the flavonoid, present in artoflavone A, cycloartobiloxanthone, and cycloartelastoxanthone, enhanced the DPPH• scavenging activity of PF derivatives. In addition, artonol A was shown to exhibit a strong concentration-dependent inhibition of the enzymatic activity of xanthine oxidase, with an IC_50_ of 43.3 ± 8.1 µM, compared to cyclogeracommunin, with an IC_50_ of 73.3 ± 19.1 µM [91].

According to Ramli et al. (2016), PF compounds artonin E, elastixanthone, and cycloartobiloxanthone, isolated from *Artocarpus elasticus* bark, displayed significant DPPH• scavenging activity according to the TLC bioautography analysis, with an IC_50_ of 11.5, 21.6, and 40.0 μg mL^−1^, respectively [50]. Ko et al. (1998) measured DPPH• decolorization induced by artonin A, artonin B, and a xanthone-like cycloheterophyllin, all isolated from *A. heterophyllus* Lam [92]. DPPH• scavenging activity was expressed as IC_0.20_, which corresponded to the concentration of the tested compounds that caused a decrease in absorbance during a given time period. The results indicated that all tested FPs enhanced DPPH• decolorization in a concentration-dependent way, with an IC_0.20_ of 8.4 ± 0.3, 12.2 ± 0.6, and 9.6 ± 0.7 μM, respectively, compared to α-tocopherol, used as a positive control (IC_0.20_ value of 11.9 ± 0.2 μM). The authors also confirmed that artonin A, artonin B, and cycloheterophyllin could scavenge the hydroxyl and peroxyl radicals generated in hydrophilic environments, and could provide good protection against lipid peroxidation when biomembranes were exposed to oxygen radicals. These FP compounds inhibited Fe(II)-induced lipid peroxidation, with IC_50_ values of 0.47 ± 0.24 µM, 0.71 ± 0.13 µM, and 0.96 ± 0.21 µM, respectively. 

Artonin A, artonin B, and cycloheterophyllin also inhibited the copper-catalyzed oxidation of human low-density lipoprotein (LDL). The authors specifically recorded the level of TBARS formation and the fluorescence spectrum of LDL exposed to oxidative conditions with or without adding the tested PF compounds. The work confirmed that artonin A, artonin B, and cycloheterophyllin inhibited both oxidative parameters in a concentration-dependent way. When added at the 30 mM concentration, they significantly suppressed Cu^2+^-induced TBARS formation with 6.0 ± 1.5, 4.5 ± 1.2, and 2.5 ± 0.8 nmol mg^−1^ protein, respectively. The estimated values came very close to those obtained for the TBARS content in unstimulated LDL (1.4 ± 0.5 nmol mg^−1^ protein) compared to the increased amount of TBARS after Cu^2+^-induced LDL oxidation (212.3 ± 21.4 nmol mg^−1^ protein). These results suggested that not only artonins A and B but also cycloheterophyllin might reach the copper-induced lipid peroxidation site and, thus, provide protection against the oxidative modification of LDL.

However, it should be emphasized that none of the three above-mentioned PFs compounds isolated from *A. heterophyllus* Lam. showed scavenging activity for superoxide anion radicals (O_2_^−^) or hydrogen peroxide (H_2_O_2_) [84].

Some other PF compounds, such as artocarpine, artocarpetin, and artocarpetin A, which were also isolated from *A. heterophyllus* Lam [93], had no effect on Fe^2+^-induced lipid peroxidation when used at the 100 µM concentration [84]. Rajendran et al. (2004) investigated artocarpin and cycloartocarpin as inhibitors of lipid peroxidation by following Mb(IV) reduction, and the results were expressed as a percentage of TBARS inhibition. The inhibition rate of MDA formation for cycloartocarpin was 34%, which was proven more efficient than artocarpin (24% inhibition) and ascorbic acid (27% inhibition) [94]. 

Cycloartocarpesin B, isolated from *A. elasticus* leaves, together with a new diprenylated dihydrochalcone elastichalcone B, exhibited DPPH• scavenging activity, with IC_50_ values of 11.89 and 11.30 μg mL^−1^, respectively [57].

Hashima et al. (2010) reported marked DPPH• scavenging activity (with an IC_50_ value of 2.0 μg mL^−1^) for pyranocycloartobiloxanthone A isolated from the stem bark of the rare endemic Malaysian *A. obtusus* species [95]. Ee et al. (2011) stated that all the xanthone-like derivatives isolated from *A. kemando* stem bark (artomandin, artoindonesianin C, and artonol B) displayed weak DPPH• scavenging activity, with IC_50_ values for the latter two FP compounds exceeding 120 μg mL^−1^. Artomandin scavenged DPPH• with an IC_50_ value of 38.0 μg ± 6.4 μg mL^−1^, which was, however, significantly lower than vitamin C (IC_50_ value of 12.2 μg mL^−1^) used as a positive control [96].

Two prenylated chalcones, 1-(2,4-dihydroxyphenyl)-3-[8-hydroxy-2-methyl-2-(4-methyl-3-pentenyl)-2H-1-benzopyran-5-yl]-1-propanone and 2-geranyl-2′,3,4,4′-tetrahydroxydihydrochalcone, from *Artocarpus altilis* showed moderate DPPH• scavenging activities compared to the catechin used as the positive control (IC_50_ of 6.5 ± 0.6 µM), with IC_50_ values of 82.2 ± 5.2 and 82.4 ± 6.1 μM, respectively. At the same time, these compounds’ NO radical scavenging activity was quite poor, with IC_50_ values close to or above 500 µM [97].

Lan et al. (2013) isolated fourteen PFs from *A. altilis* cortex and heartwood, including seven flavones (artocarpin, artoflavone A, hydroxyartoflavone A, artogomezianone, 10-oxoartogomezianone, 8-geranyl-3-(hydroxyprenyl)isoetin, and 8-geranylapigenin) and seven xanthone-like compounds (cyclocommunol, cyclo-artocarpin, cyclogeracommunin, isocycloartobiloxanthone, cyclomorusin, cudraflavone A, and artonin M) to determine their DPPH• scavenging activity. Most of the tested compounds possessed rather poor scavenging activity (IC_50_ > 300 μM), except for hydroxyartoflavone A (IC_50_ of 20.9 ± 2.1 μM), isocycloartobiloxanthone (IC_50_ of 33.9 ± 1.5 μM), and artoflavone A (IC_50_ of 53.5 ± 3.1 μM). However, they were all weaker free radical scavengers than the positive control quercetin (with an IC_50_ value of 10.2 ± 1.4 μM) [98].

Of the above-mentioned flavonoids from *A. altilis*, only five compounds were further analyzed by means of an ABTS^+^ scavenging assay [98]. The most efficient scavenger was isocycloartobiloxanthone, with an IC_50_ of 7.2 ± 1.6 µM, which came close to the value of the positive control quercetin (IC_50_ 7.8 ± 2.1 µM). The other investigated PFs, such as artogomezianone (IC_50_ of 36.9 ± 2.3 µM), 8-geranyl-3-(hydroxyprenyl)isoetin (IC_50_ value of 156.9 ± 5.3 µM), and artocarpin (IC_50_ of 265.1 ± 4.3 µM), exhibited significantly lesser scavenging efficiency, with no activity found for cyclocommunol (IC_50_ value above 500 µM).

The same group of authors also investigated the activity of some flavonoids isolated from *A. altilis* for superoxide anion-scavenging. They concluded that artogomezianone, norartocarpetin, and artocarpin had a moderate scavenging potential compared to quercetin (IC_50_ 3.9 ± 0.4 µM), with IC_50_ values of 39.7 ± 3.3 µM, 74.1 ± 5.1 µM, and 94.1 ± 1.8 µM, respectively. However, the O_2_^-^ scavenging activity percentage for 8-geranyl-3-(hydroxyprenyl)isoetin, isocycloartobiloxanthone, and cyclocommunol was quite low, with IC_50_ values over 300 µM [98].

Two new PFs, flavon-4′,5-dihydroxy-6,7-(2,2-dimethylpyrano)-2′-methoxy-8-γ,γ-dimethylallylflavone and xanthone-3′-hydroxycycloartocarpin, together with six other PFs (5,7-dihydroxy-4′-methoxy-8-prenylflavanone, isobavachalcone, pyranocycloartobiloxanthone A, artocarpin, chaplashin, and cycloartocarpin), were isolated from *A. anisophyllus* heartwood and leaves, and subsequently examined for their antioxidant activities [87]. Pyranocycloartobiloxanthone A, artocarpin, and 3′-hydroxycycloartocarpin exhibited DPPH• scavenging activity, with SC_50_ values (concentration producing 50% of maximal stimulation in 30 min) of 20.2 and 140.0 152.9 μg mL^−1^, respectively. In contrast, cycloartocarpin possessed no scavenging activity, while isobavachalcone was a poor DPPH• scavenger, with an SC_50_ value above 400 μg mL^−1^. The SC_50_ value of butylated hydroxyanisole (BHA), used as a standard reference, was 17.5 μg mL^−1^ [87]. 

Four flavone-like PFs (artocarpin, 4′,5-dihydroxy-6,7-(2,2-dimethylpyrano)-2′-methoxy-8-(γ,γ-dimethyl)allyflavone, artonin E, and macakurzin C), two xanthones (cudraflavone A and cycloartobiloxanthone), and one chalcone (flemichapparin A) were isolated from *A. scortechinii* King leaves and stem bark, and subsequently analyzed for their DPPH• scavenging activity [88]. The results showed that flemichapparin A, artonin E, and cycloartobiloxanthone obtained IC_50_ values of 131.0, 151.6, and 196.0 μM, respectively. However, it was not possible to determine the IC_50_ values of the other isolated compounds. Furthermore, all the experimentally measured IC_50_ values lay between the two positive controls used in this assay: BHT, as a significantly weaker scavenger (IC_50_ value of 231.6 μM), and the much stronger scavenger BHA, with an IC_50_ value of 49.87 μM.

Of the seven PFs isolated from *A. scortechinii* King, it was possible to evaluate only the ABTS^+^ scavenging potential of artonin E (IC_50_ of 145.0 µM), flemichapparin A (IC_50_ of 199.7 µM), and cycloartobiloxanthone (IC_50_ of 269.0 µM). The positive control BHA was a stronger scavenger than all the analyzed compounds, with an IC_50_ value of 91.01 µM [88].

Zakaria et al. (2017) isolated the xanthone tephrosin and two flavones, cudraflavone C and artocarpin, from *A. integer* (Thunb.) Merr heartwood and determined their DPPH• scavenging activity. Tephrosin was a poor scavenger, with an IC_50_ value of 55.58 μg mL^−1^, compared to the control (IC_50_ of ascorbic acid was 2.79 μg mL^−1^). In contrast, both flavones were almost 10-fold more effective, with IC_50_ values of 3.35 μg mL^−1^ and 4.70 μg mL^−1^, respectively [99].

Two prenylated flavones (cycloartocarpesin B and cudraflavone B) and three flavanones (euchrestaflavanone B, euchrestaflavanone C, and an unknown flavanone), isolated from *Cudrania tricuspidata* (Carr.) Bureau root bark, showed no significant DPPH• scavenging activity (IC_50_ > 300 μM). However, all PFs strongly scavenged the ABTS radical, with IC_50_ values ranging from 4.2 ± 1.3 µM (for cudraflavone B) to 8.3 ± 0.8 µM (for a new PF), and were similar to those of the control compound quercetin (4.0 ± 1.6 µM). Moreover, all the above-mentioned compounds displayed effective scavenging activity against the hydroxyl radical in a hydrophilic environment as measured by means of ESR spectroscopy. In particular, the radicals generated by a Fenton reaction were trapped as DMPO-OH spin adducts, and their signal intensity was reduced in the presence of radical scavengers. The IC_50_ values determined for PFs fell within a concentration range from 40.6 ± 2.1 to 75.3 ± 4.8 µM, while the hydroxyl radical scavenging activity of Trolox was 48.2 ± 2.3 µM [100].

The PFs isolated from *C. tricuspidata* were further tested for their potential to inhibit the Cu^2+^-induced oxidation of LDL. The results revealed all the compounds’ moderate antioxidant activity against LDL oxidation, with IC_50_ values ranging from 27.2 to 65.6 µM, determined by measuring the amount of TBARS when using probucol as a positive control (with an IC_50_ of 3.6 µM) [101].

Prenylated flavonol 6-prenylated-3,5,7,4′-tetrahydroxy-2′-methoxyflavonol, with remarkable antioxidant properties, was isolated from *Chlorophora regia* stem bark. This compound exhibited significant DPPH radical scavenging activity compared to the standard Trolox (IC_50_ of 1.1 μg mL^−1^), with an IC_50_ value of 2.8 μg mL^−1^ [102].

Three PFs, flavanones 6,8-diprenyleriodictyol and dorsmanin F and flavonol dorsmanin C, were isolated from medicinal plant *Dorstenia mannii* twig and leaves, and tested for their antioxidant activity at the 1, 10, and 100 μM concentrations. All the investigated compounds were strong DPPH• scavengers in a concentration-dependent way, and were even stronger than butylated hydroxytoluene (BHT). Nevertheless, flavonol dorsmanin C possessed the greatest scavenging activity, which highlighted the importance of the C2=C3 double bond and the 3-OH group of the flavonol for having a high scavenging potential compared to flavanones [103]. The PFs from *Dorstenia mannii* were also effective inhibitors of the Cu^2+^-catalyzed oxidation of LDL, with IC_50_ values of 1 μM, which were similar to the inhibitory effect of the non-PF quercetin. No prooxidant activity occurred when high concentrations of 6,8-diprenyleriodictyol, dorsmanin F, and dorsmanin C were used. Apparently, such lack of prooxidant activity might contribute significantly to prenylated compounds’ overall biological activity [103].

Omisore et al. (2005) tested the DPPH• scavenging activity of extracts and/or flavonoid compounds from some *Dorstenia* species, such as 6-prenyl-apigenin from *D. kameruniana*, and two flavanones, 6,8-diprenyleriodictyol and dorsmanin F, isolated from *D. mannii*. According to the results, 6,8-diprenyleriodictyol showed good antioxidant capacity, with a half-maximal effective concentration EC_50_ of 32.12 ± 1.10 μg mL^−1^, as did control compounds ascorbic acid (EC_50_ of 19.33 ± 0.3 μg mL^−1^) and quercitrin (EC_50_ 28.16 ± 0.84 μg mL^−1^). Compounds 6-prenylapigenin, with an EC_50_ value of 86.43 ± 0.26 μg mL^−1^, and dorsmanin F (EC_50_ value of 53.89 μg mL^−1^) exerted quite moderate scavenging effects [89].

Two new PFs, namely, thonningiol and thonningiisoflavone, were isolated from *Ficus thonningii* together with other known compounds, and structurally characterized by Fongang et al. (2015). The antioxidant assay confirmed that thonningiisoflavone exhibited good DPPH• scavenging activity, with an IC_50_ value of 65.50 ± 0.44 µM, compared to butylated hydroxyanisole (BHA) used as a standard compound (IC_50_ 44.20 ± 0.32 µM) [104].

Iso-PF morusin is abundant in *M. alba* roots, but also appears in other Moraceae species. According to Martins et al. (2021), morusin extracted from the roots of different *M. alba* varieties showed weak free radical scavenging activity, with IC_50_ values of 1819.83 ± 144.53 µM and 297.83 ± 7.27 µM for DPPH and ABTS assays, respectively [105]. The antioxidant activities of the PF compounds of the Moraceae family are shown in Table 1.

### 3.5. Anti-Inflammatory Activity

Inflammation is a complex process in the human body. It involves a number of inflammatory mediators, such as reactive oxygen species (ROS), interleukin (IL)-1β, tumor necrosis factor-α (TNF-α), IL-6, nitric oxide (NO), NF-k, etc. [106]. Despite numerous drugs on the market that treat inflammation via a variety of different mechanisms, it is still unfeasible to successfully treat inflammatory disorders. Therefore, new active substances must be developed whose mechanism of action differs from those of conventional anti-inflammatory drugs. Interestingly, PFs and their derivatives with anti-inflammatory activities have been repeatedly reported [107]. In this context, several PF compounds and their derivatives from Moraceae family were obtained (broussonol D, morusin, atalantoflavone, sanggenon B, sanggenon D, and cudraflavone B), and their anti-inflammatory effect was subsequently tested [108,109,110,111,112,113,114]. The results showed that most PFs possessed moderate anti-inflammatory activity in LPS-stimulated RAW 264.7 cells by down-regulating COX-2 induction at 10–25 μM. In particular, cudraflavone B, which contains a cyclized prenyl group on ring A and bears an additional 2′,4′-OH moiety on ring B, exhibited the most potent anti-inflammatory activity by inhibiting COX-2 activity (with an IC_50_ value of 2.5 μM). This effect was comparable to that of the common COX inhibitor indomethacin (IC_50_ of 1.9 μM), showing that the cudraflavone B was a potent COX-2 inhibitor and had some potential as a new non-steroidal anti-inflammatory drug. It was reported that 5,7,4′-trihydroxy-6-geranylflavanone, a PF isolated from the fruits of *Artocarpus communis*, showed great anti-inflammatory activity in vitro [115]. According to the study, a low concentration of 5,7,4′-trihydroxy-6-geranylflavanone (≤2.5 μM) had a strong inhibitory effect on the RAGE gene expression, and down-regulated both TNF-α and IL-1β secretion and the expression of genes in THP-1 cells. In addition, the production of pro-inflammatory cytokine TNF-α in LPS-stimulated macrophages was used for the determination of the anti-inflammatory activity of selected PFs from Moraceae plants (kuwanon C, kuwanon E, sanggenon H, morusinol, soroceal, sanggenon E, and kuwanon C) [52,108]. The outcome indicated that compounds kuwanon E, sanggenon H, morusinol, and soroceal were the most efficient ones in significantly inhibiting TNF-α secretion. Additionally, compound morusinol (IC_50_ < 10 μM) displayed the strongest inhibitory effect, which was nearly twice that of the positive control prednisone. 

Nitric oxide (NO) is a short-lived radical and cellular signaling molecule formed from L-arginine catalyzed by nitric oxide synthase (NOS) [116]. An inducible NOS produces high nitric oxide levels, which plays an important role in the regulation of immunomodulatory and inflammatory responses [117,118]. However, excessive NO production may result in inflammatory diseases in the immune system. Therefore, the inhibition of NO production opens a new avenue to search for potential anti-inflammatory drugs. 

A series of PFs with inhibitory effect on lipopolysaccharide-induced NO production was isolated from the heartwood of *Artocarpus communis* [119]. Of the compounds tested, isobacachalcone and gemichalcone B showed the strongest inhibitory effect on NO production in RAW 264.7 LPS-activated mouse macrophage cells, with IC_50_ values of 6.4 µM and 9.3 µM, respectively. In addition, 3″,3″-dimethylpyrano[3′,4′]2,4,2′-trihydroxychalcone, morachalcone A, artocarpin, and (2S)-euchrenone a_7_ also showed high inhibitory activity, with IC_50_ values ranging from 12.3 µM to 18.8 µM, while cudraflavone C and the flavonoid derivatives (+)-cycloartocarpin and (+)-cudraflavone A were weak inhibitors of NO production (IC_50_ values above 40 µM).

Liu et al. (2019) investigated prenylated isoflavone derivatives ficucaricones A–D, isolated from fruits of *F. carica*, for their anti-inflammatory effects on lipopolysaccharide-induced NO production in RAW 264.7 mouse macrophage cells in vitro by measuring their inhibitory effects. The results showed that the tested prenylated isoflavone derivatives exhibited strong inhibitory activity against NO production, with IC_50_ values ranging from 0.89 ± 0.05 to 3.29 ± 0.12 μM for ficucaricone A and C, respectively. The authors concluded that the anti-inflammatory effect of the tested compounds was correlated with their chemical structures. A detailed analysis revealed that the substitution pattern of the benzene ring A of ficucaricone C could play an important role in the strong inhibition of NO production. In addition, ficucaricone D with a para-disubstituted benzene ring was shown to be more likely to have a strong inhibitory effect on NO production comparable to that of hydrocortisone [120].

β-hexosaminidase is a chemical mediator defined as the parameter of mast cell degranulation [118]. Mast cell degranulation leads to an acute response, such as increased vascular permeability, vasodilation, and inflammation. [121]. Therefore, β-hexosaminidase was considered as an indicator for the estimation of mast cell activation in various allergic inflammations. In addition, inhibiting the release of β-hexosaminidase would be a useful way to treat inflammation. A number of PF derivatives (derrone, alpinumisoflavone, 4′-O-methylalpinumisoflavone, erysenegalensein E, 5,7,3′,4′-tetrahydroxy-6,8-diprenylisoflavone, 5,7,4′-trihydroxy-6,8-diprenylisoflavone, isoerysenegalensein E, gancaonin A, warangalone, and 2-[{3-hydroxy-2′,2-dimethyl-8-(3-methyl-2-butenyl)}chroman-6-yl]-7-hydroxy-8-(3-methyl-2-butenyl)-chroman-4-one) were isolated from the fruits of *Cudrania tricuspidata* and tested for their inhibitory effects on β-hexosaminidase [121]. The results showed that, except for compound 5,7,3′,4′-tetrahydroxy-6,8-diprenylisoflavone, which inhibited the release of β-hexosaminidase in FcεRI-mediated mast cells with an IC_50_ value of 20.4 μM, the tested PFs had no obvious inhibitory effect when compared with control compound ketotifen (IC_50_ of 23.1 μM).

All the above-mentioned results indicated that the relationship between the structure of PFs and anti-inflammatory activity pointed to the following: (a) the keto group at the C-4 position and the hydroxy groups at C-5, C-7 and C-4′ positions of the prenyl flavonoid skeleton represent significant structural segments for anti-inflammatory activity; (b) the double bond between C-2 and C-3 does not significantly influence anti-inflammatory activity (kuwanon C); (c) the cyclization of the prenyl group in C-6 or C-8 may also contribute to the inhibitory activity against COX, TNF-α, and NO production (cudraflavone B and morusinol); (d) two prenyl side chains located on rings A and C have the strongest anti-inflammatory effect (kuwanon C, cudraflavone B, and morusinol); (e) the prenylated isoflavones possess notable inhibitory activity against NO production; and (f) the methoxy group at C-7 position is favorable for the activity increment (ficucaricone D and 4′-hydroxy-5,7-dimethoy-6-(3-methyl-2-buteny)-isoflavone). Table 1 summarizes the anti-inflammatory activity of PF compounds of the Moraceae family.

### 3.6. Enzyme Inhibition Activity

The loss of melanin and depigmentation can be serious esthetic and dermatological problems. On the contrary, this pigment’s increased synthesis and accumulation are associated with numerous skin diseases. Tyrosinase and tyrosinase-related enzymes play a crucial role in the biosynthesis of melanin-melanogenesis [122]. Conversely, inhibitors of tyrosinase represent the key components in the regulation of excessive melanin production. To date, tyrosinase inhibitors that derive from natural resources have attracted more interest than their synthetic structural analogs.

According to Arung et al. (2006), natural polyphenolic flavone compounds with prenyl substituents could be potentially used for skin lightening and against hyperpigmentation. Isoprenoid-substituted flavonoids isolated from *Artocarpus heterophyllus* wood, such as artocarpin, cudraflavone C, 6-prenylapigenin, kuwanon C, norartocarpin, and albanin A, inhibited melanin biosynthesis in B16 melanoma cells without inhibiting tyrosinase activity (IC_50_ values > 300 µM). In addition, the depigmenting mechanism involved the suppression of pigmentation signals rather than the inhibition of tyrosinase activity [123].

In related studies by the same group, all the above PFs, together with brosimone I and cudraflavone B, were found to inhibit melanin production significantly more than kojic acid and arbutin, with IC_50_ values falling within the 0.8–7.3 µM range [124]. These authors revealed that the presence of an isoprenoid-substituted moiety in the tested PFs notably enhanced their inhibitory effect on melanin biosynthesis. However, the presence of a prenyl side chain at C(3) reduced the flavonoids’ potential to inhibit tyrosinase activity [124].

Lan et al. (2013) investigated whether five new prenylated flavones, together with some other known PF compounds isolated from *A. altilis* bark and heartwood, could act as natural tyrosinase inhibitors. The results showed that norartocarpetin, artogomezianone, cudraflavone A, and artonin M strongly suppressed tyrosinase activity and thus inhibited melanin production. Conversely, artocarpin lowered melanin content, but did not inhibit tyrosinase. Norartocarpetin strongly inhibited tyrosinase activity, with an IC_50_ of 0.42 ± 1.1 µM, while artonin M, artogomezianone and cudraflavone A showed a weaker inhibitory effect, with IC_50_ values of 74.1 ± 2.2, 84.8 ± 5.8 and 88.4 ± 6.2 µM, respectively. In addition, some tested PF compounds, such as cycloartobiloxanthone, artocarpin, and cyclocommunol, were as effective tyrosinase inhibitors as the control arbutin [98].

Similar results were previously obtained by Shimizu et al. (2002). When investigating the inhibitors of melanin biosynthesis, the authors concluded that artocarpin, as the main constituent of the *Artocarpus incises* L. heartwood extract, evoked a remarkable skin-lightening effect, but no inhibitory effect on tyrosinase [125].

Nguyen et al. (2017) investigated the tyrosinase inhibitory effect of an *Artocarpus rigida* stem extract and concluded that norartocarpetin showed a very strong anti-tyrosinase effect among all tested PFs, with an IC_50_ value of 0.023 μM, compared to the standard compound kojic acid (IC_50_ of 44.6 μM) [126].

The tyrosinase inhibitory activity of the PFs isolated from *Artocarpus chama* stem was investigated by Dej-adisai et al. (2022) [127]. One of the new compounds, identified as 3′-farnesyl-apigenin, had moderate anti-tyrosinase activity with an IC_50_ of 135.70 ± 1.16 µg mL^−1^, while 3-(hydroxyprenyl)-isoetin and 3-prenyl-5,7,2′,5′-tetrahydroxy-4′-methoxyflavone (with IC_50_ values above 200 µg mL^−1^) were almost ineffective as tyrosinase inhibitors. Of all tested PFs isolated from *A. chama*, artocarpanone displayed the most marked activity against tyrosinase, with similar IC_50_ values to the control values (38.78 ± 1.02 µg mL^−1^).

PF broussochalcone A and kazinol B, isolated from *Broussonetia papyriferra* (L.) Vent. root bark, had no appreciable inhibitory activity against tyrosinase (with 7% and 10% inhibition, respectively, at the 100 µM concentration). In contrast, papyriflavonol A much more efficiently inhibited tyrosinase, with activity values close to those of quercetin (about 40%) [128].

According to Park et al. (2017), PFs isolated from *B. papyriferra* roots possessed antiviral activities against CoV cysteine proteases and could be considered as a valuable source of anticoronavirus agents. The results confirmed that all PFs tested were more effective against SARS-CoV papain-like protease (PL^pro^) compared to the other cysteine proteases used (3-chymotripsin-like protease, 3CL^pro^), with IC_50_ values ranging from 3.7 ± 1.6 µM (for papyriflavonol A) to 66.2 ± 6.8 µM (for kazinol A). In addition, broussochalcone B significantly inhibited the activity of MERS-CoV3CL^pro^, with an IC_50_ of 27.9 ± 1.2 µM. On the other hand, a rather low inhibitory potential of the tested PFs against MERS-CoV PL^pro^ cysteine proteases was observed [101].

Lee et al. (2001) [129] investigated the inhibitory effect of PF compounds isolated from *B. papyrifera* on aromatase, an enzyme involved in estrogen biosynthesis. The results revealed that 2S-2′,4′-dihydroxy-2″-(1-hydroxy-1-methylethyl)dihydrofuro-[2,3-h]-flavanone and 3′-[γ-hydroxymethyl-(E)-γ-methylallyl]-2,4,2′,4′-tetrahydroxychalcone11′-O-coumarate represented potent aromatase inhibitors, with IC_50_ values of 0.1 µM and 0.5 µM, respectively. Moreover, the inhibitory activity of isogemichalcone C came close to the positive control values (IC_50_ of 7.5 µM), while 5,7,2′,4′-tetrahydroxy-3-geranylflavone showed weak anti-aromatase activity [129].

Hu et al. (2012) analyzed *Morus yunnanensis* leaf extract and concluded that two new PFs, morusyunnansins E, (2S)- and morusyunnansins F, together with 2′,4′-dihydroxy-7-methoxy-8-prenylflavan, were potent tyrosinase inhibitors, with IC_50_ values of 1.43 ± 0.43 μM, 0.15 ± 0.04 μM, and 0.81 ± 0.17 μM, respectively. These authors also emphasized that morusyunnansins F showed approximately 200-fold greater activity than kojic acid [130].

The extract from *Morus lhou* (S.) Koidz. roots is a proven source of PFs with variable tyrosinase inhibitory activity [131]. In particular, 8-isoprenyl-5′-geranyl-5,7,2′,4′-tetrahydroxy flavanone and kuwanon E featured among the most potent inhibitors of monophenolase activity, with IC_50_ values of 44.2 ± 0.9 µM and 47.5 ± 4.0, respectively. Neocyclomorusin (IC_50_ 127.4 ± 3.9 µM) and kuwanon A (IC_50_ 131.8 ± 10.6 µM) showed a moderate inhibitory effect, while kuwanon U and morusinol exhibited very poor activity (IC_50_ above 200 µM).

Lee et al. (2004) studied the inhibitory effect of kuwanon C and morusin, extracted from *Morus alba* L root bark, and sanggenon D, from *Morus mongolica* Schneider root bark, against tyrosinase. Their results demonstrated remarkable concentration-dependent inhibition by sanggenon D and kuwanon C, with IC_50_ values of 7.3 µM and 49.2 µM, respectively, but morusin exhibited no appreciable inhibitory effect [132].

The tyrosinase inhibitory activities of the constituents isolated from *Morus alba* L. leaves revealed that euchrenone a_7_ (IC_50_ 0.260 ± 0.026 µM), moracin N (IC_50_ 0.924 ± 0.048 µM), moracin C (IC_50_ 2.27 ± 0.44 µM), and chalcomoracin (IC_50_ of 26.8 ± 2.5 µM) were the most potent inhibitors compared to standard compound kojic acid (IC_50_ 15.9 ± 2.1 µM) [31].

Bioactive compounds from *M. alba* twigs were also considered a promising natural source for producing various whitening and anti-hyperpigmentation agents [133]. Morachalcone A, with an IC_50_ value of 0.08 ± 0.02 µM, had a very strong tyrosinase inhibitory effect compared to kojic acid, while the activity of kuwanon C (IC_50_ 52.00 ± 2.50 µM) and cyclomulberrin (IC_50_ of 66.30 ± 7.20 µM) came close to the control values.

Guo et al. (2018) reported many natural inhibitors of phosphodiesterases (PDEs) with different structures, including a series of PFs isolated from *M. alba* root bark. According to their results, most PF compounds displayed potent inhibitory activity against phosphodiesterase-4 (PDE4), with IC_50_ values ranging from 0.0054 ± 0.0003 μM (detected for (±)-cyclomorusin) to 1.88 ± 0.10 μM (obtained for 5′-(1″,1″-dimethylallyl)-5,7,2′,4′-tetrahydroxyflavone). Interestingly, 50% of all tested PFs showed higher inhibitory activity than rolipram used as the control (with IC_50_ < 0.6 μM). The molecular dynamics simulations performed by the same authors revealed that the presence of prenyl moieties and free hydroxyl groups in PFs’ structure could be extremely important for their remarkable activity against PDEs [134]. Table 1 summarizes the enzyme inhibition activity of the PF compounds of the Moraceae family.

### 3.7. Anti-Alzheimer and Neuroprotective Activity

Alzheimer’s disease (AD) is a neurodegenerative disorder of the brain characterized by progressive deficits in memory and cognitive/behavioral impairments that eventually lead to dementia [135]. The inhibition of acetylcholinesterase (AChE), butyrylcholinesterase (BChE), and β-site amyloid precursor protein cleaving enzyme 1 (BACE1) was found to play a critical role in the prevention and treatment of AD. Kuk et al. (2017) investigated the anti-AD activity of PF compounds isolated from the root bark of *M. alba*, including mulberrofuran G, albanol B, and kuwanon G [136]. The results showed that mulberrofuran G and albanol B were potent AChE inhibitors, with IC_50_ values of 2.13 ± 0.02 µM and 2.47 ± 0.06 µM, respectively. All PFs tested significantly inhibited BChE compared to the control compound berberine, with albanol B (IC_50_ value of 1.39 ± 0.06 µM) being the most effective. On the other hand, mulberrofuran G showed the strongest BACE1 inhibitory activity of all PFs tested (IC_50_ of 0.31 ± 0.01 µM).

Kim et al. (2011) focused on the AChE- and BChE-inhibitory activity of PFs isolated from the root bark of *Morus lhou* L. (5′-geranyl-4′-methoxy-5,7,2′-trihydroxyflavone, 5′-geranyl-5,7,2′,4′-tetrahydroxyflavone, kuwanon U, kuwanon E, morusin, morusinol, cyclomorusin, neocyclomorusin, and kuwanon C). Except for morusinol, all the PFs compounds tested inhibited the cholinesterase enzymes in a dose-dependent manner, with IC_50_ values between 10.95 μM and 36.4 μM, and between 3.43 μM and 24.08 μM against AChE and BChE, respectively. Moreover, the inhibitory effect of 5′-geranyl-4′-methoxy-5,7,2′-trihydroxyflavone was even stronger than that of the control compound eserin (IC_50_ of 4.72 μM for BChE). The authors emphasized that the presence of a free hydroxyl group at C-7, as a component of the 5,7-dihydroxyphenol motif on the A ring, could be important for the activity of the PFs. Furthermore, it was found that methylation at the C4′-OH only slightly effected the inhibitory activity of the PF compounds, as seen with 5′-geranyl-4′-methoxy-5,7,2′-trihydroxyflavone and 5′-geranyl-5,7,2′,4′-tetrahydroxyflavone or kuwanon U and kuwanon E. Conversely, the hydration of the prenyl group at C-3 significantly reduced the inhibitory effect of PFs, as shown for morusin and morusinol. This also suggested that hydrophobicity was important for the inhibitory effect of alkylated flavonoids from *M. lhou* [137].

Among the numerous compounds isolated from the root bark of *Cudrania tricuspidata*, cudraflavone B was recognized as a potential therapeutic agent for neurodegenerative diseases. This naturally occurring PF compound showed neuroprotective effects and the inhibition of reactive oxygen species (ROS) against glutamate-induced neurotoxicity in HT22 mouse hippocampal cells [138]. Glutamate treatment increased HT22 cell death, while cudraflavone B at non-cytotoxic concentrations (20 and 40 μM) increased cell viability in a dose-dependent manner, with an EC_50_ value of 23.1 ± 3.7 μM. In addition, cudraflavone B strongly suppressed glutamate-induced ROS production, with an EC_50_ value of 19.4 ± 4.1 μM [138].

In addition, Ko et al. (2020) confirmed the neuroprotective activity of kuwanon C, isolated from the roots of *C. tricuspidata* in HT22 cells, as well as the anti-neuroinflammatory effects of this PF compound in BV2 cells via Nrf2-mediated HO-1 regulation [139].

Hiep et al. (2015) showed that the prenylated isoflavones cudraisoflavone H, cudraisoflavone I, cudraisoflavone J, 5,7,3′,4′-tetrahydroxy-6,8-diprenylisoflavone, erythrinin B, and gancaonin B, isolated from the fruits of *C. tricuspidata*, had a high therapeutic potential for the treatment of neurotoxicity [140]. These PFs showed significant neuroprotective activity against 6-hydroxydopamine (6-OHDA)-induced cell death in human neuroblastoma cells SH-SY5Y, with EC_50_ values ranging from 0.5 ± 0.09 µM to 9.2 ± 0.07 µM for cudraisoflavone J and cudraisoflavone I, respectively. In addition, it was reported by Kwon et al. (2016) that cudraflavone A, isolated from the root bark of *C. tricuspidata*, had moderate neuroprotective effects against 6-OHDA-induced cell death, with an IC_50_ value of 15.5 µM. The same authors also showed that cudratrixanthones D, E, H–K, M, and N, 3-*O*-methyl-cudratrixanthone G, cudraxanthone D, 3-*O*-demethylcudraxanthone B, and gerontoxanthone C had various neuroprotective activities against oxygen-glucose deprivation (OGD)-induced cell death, with EC_50_ values in the range of 2.9 μM (3-*O*-methylcudratrixanthone G) to 34.9 μM (cudratrixanthone H), compared to control carnosine (EC_50_ of 3.0 μM). Conversely, the results showed that only cudratricusxanthone J among the PF compounds tested had neuroprotective effects against cell death induced by 1-methyl-4-phenylpyridinium ions (MPP+), with an EC_50_ value of 8.1 μM [141].

Neurodegenerative disorders are mainly associated with neuroinflammation [142]. Under inflammatory conditions, microglial cells induce the production of proinflammatory mediators such as nitric oxide (NO), prostaglandin E2 (PGE2), inducible nitric oxide synthase (iNOS), cyclooxygenase (COX)-2, pro-inflammatory cytokines like interleukin (IL)-1b, IL-6, and tumor necrosis factor (TNF)-a. Therefore, substances that inhibit their release could be useful for the treatment of neuroinflammatory diseases. According to the study by Kim et al. (2018), cudraflavanone A, isolated from the root bark of *C. tricuspidata*, showed an anti-neuroinflammatory effect against LPS-induced inflammation in BV2 microglial cells. This PF compound significantly inhibited NO and PGE2 production, with IC_50_ values of 22.2 µM and 20.6 µM for NO and PGE2, respectively. From this, the authors concluded that cudraflavanone A could serve as an effective substrate for the development of therapeutics against neurodegenerative diseases [143].

Ferroptosis is a novel form of regulated cell death associated with degenerative disorders and is triggered by erastin [144]. Wen et al. (2020) investigated the effects of morachalcone D and morachalcone E, isolated from the leaves of *M. alba*, on erastin-induced ferroptosis and concluded that this process was attenuated by morachalcone D in HT22 cells in a dose-dependent manner (15–50 μM) [145]. Moreover, the viability of HT22 cells treated with erastin and morachalcone D was significantly higher (15.10%) compared to control, ranging from 17.65% to 76.36% at the tested concentrations, with an EC_50_ value of 33.73 ± 0.89 μM. In contrast, the treatment of HT22 cells with morachalcone E resulted in a rather small increase in cell viability (0–22.15%), suggesting that morachalcone D was much more active in suppressing ferroptosis, and could be used as a safe ferroptosis inhibitor. The authors hypothesized that the difference in the prenyl pattern of morachalcone D and morachalcone E might be related to their different effects on erastin-induced ferroptosis. The anti-Alzheimer and neuroprotective activity of PF compounds of the Moraceae family is summarized in Table 1.

## 4. Conclusions

This review comprehensively summarizes the research progress on the biosynthesis and biological activities of PFs. Compelling evidence indicates that prenylation could increase the bioactivities of their backbone flavonoids. Therefore, PFs have the potential to be developed as new drugs or dietary supplements. To date, few PFs have been clinically investigated. Thus, PFs offer good clues for drug discovery, which provides the opportunity to design more selective and active isoprene-based PFs to treat multifactorial diseases. In addition, future studies need to explore the underlying action mechanisms and clinical studies of PFs.

## Figures and Tables

**Table 1:** Biological activities of PFs found in plants from the Moraceae family.

Compounds	Measured Parameters	Species	Biological Activities							References
12-hydroxyartonin E	IC_50_ = 0.9 µg mL^−1^	*Artocarpus rigida*			AM					[66]
Cudraflavone A	Tyrosinase inhibition: IC_50_ = 88.4 µM	*Artocarpus altilis*						EI		[98]
(2R)-Cudraflavanone H	Against MPP+-induced cell death: EC_50_ > 50 µM	*Cudrania tricuspidata*							AA	[141]
	Against OGD-induced neurotoxicity: EC_50_ > 50 µM								AA	
	Against 6-OHDA-induced cell death: EC_50_ > 50 µM								AA	
(2R)-ficusflavonoid A	Hela, MCF-7, HepG2, H460: IC_50_ > 40 µM	*Ficus hirta*		AC						[146]
(2R)/(2S)-euchrenone a_7_	IC_50_ = 6.28 ± 1.01 µM	*Morus alba*	AD							[31]
(2S)-2′,4′-dihydroxy-7-methoxy-8-prenylflavan	Tyrosinase inhibition: IC_50_ = 0.81 µM	*Morus yunnanensis*						EI		[130]
(2S)-7,2′-dihydroxy-4′-methoxy-8-prenylflavan	Tyrosinase inhibition: IC_50_ > 100 µM							EI		[130]
(2S)-7,4′-dihydroxy-8-prenylflavan	Tyrosinase inhibition: IC_50_ > 100 µM							EI		[130]
(2S)-cudraflavanone H	Against MPP+-induced cell death: EC_50_ > 50 µM Against OGD-induced neurotoxicity: EC_50_ > 50 µM Against 6-OHDA-induced cell death: EC_50_ > 50 µM	*Cudrania tricuspidata*							AA	[141]
(2S)-Ficusflavonid A	Hela, MCF-7, HepG2, H460: IC_50_ > 40 µM	*Ficus hirta*		AC						[146]
4ʹ-hydroxy-8-methoxy-6-(4-methyl-1H-pyrrol-2-yl)-flavone	C_50_ = 51.5 μg mL^−1^ IR = 88.3% ± 6.2 µM	*Artocarpus champeden*			AM					[62]
10-Oxoartogomezianone	Tyrosinase inhibition: IC_50_ > 300.0 µM	*Artocarpus altilis*						EI		[98]
14-hydroxyartonin E	ND	*Artocarpus lanceifolius*			AM					[61]
2-[{3-hydroxy-2′,2-dimethyl-8-(3-methyl-2-butenyl)}chroman-6-yl]-7-hydroxy-8-(3-methyl-2-butenyl)-chroman-4-one	Inhibition of β-hexosaminidase release: IC_50_ > 40 µM	*Cudrania tricuspidata*					AI			[121]
2″,2″-dimethylpyran-(5″,6″:6,7)-5,4′-dihydroxy-4′-methoxy-flavonol	IR = >65%	*Artocarpus champeden*			AM					[62]
3′-(3-methylbut-2-en-1-yl) biochanin A	IC_50_ = 22.1 ± 7.6 µM	*Ficus hispida*	AD							[28]
3′-(3-methylbut-2-enyl)-3′,4′,7-trihydroxyflavane	IC_50_ = 75.7 ± 2.0 µM	*Broussonetia papyrifera*	AD							[25]
3′-geranyl-3-prenyl-2′,4′,5,7-tetrahydroxyflavone	HeLa: IC_50_ = 1.32 ± 0.51 µM MCF-7: IC_50_ = 3.92 ± 0.91 µM	*Morus alba*		AC						[56]
4-hydroxylonchocarpin	MIC = 1.2 µg mL^−1^ MIC = 16 μg mL^−1^	*Dorstenia barteri*			AM					[71]
4′,5,7-trihydroxy-6-[(1R*,6R*)-3-methyl-6-(1-methylethenyl)cyclohex-2-en-1-yl] isoflavone	ND	*Ficus hispida*	AD							[28]
4′-hydroxy-5,7-dimethoxy-6-(3-methyl-2-buteny)-isoflavone	Inhibition of NO production: IC_50_ = 2.46 µM	*Ficus carica*					AI			[28]
4′-*O*-methylalpinumisoflavone	Inhibition of β-hexosaminidase release: IC_50_ > 40	*Cudrania tricuspidata*					AI			[62]
4′-hydroxy-8-methoxy-6-(4-methylfuran-2-yl)-flavone)	IR = 65%	*Artocarpus champeden*			AM					[62]
5,7-dihydroxy-6-(2″-hydroxy-3″-methylbut-3″-enyl)-4′-methoxylisoflavone	ND	*Maclura tricuspidata*	AD							[30]
5,7,3′,4′-tetrahydroxy-6,8-diprenylisoflavone	Inhibition of β-hexosaminidase release: IC_50_ = 20.4 µM	*Cudrania tricuspidata*					AI			[62]
5,7,4′-trihydroxy-6-geranylflavanone	Inhibition of TNF-α and IL-1β: IC_50_ ≤ 2.5 µM	*Artocarpus communis*					AI			[115]
5,7,4′-trihydroxy-6,8-diprenylisoflavone	Inhibition of β-hexosaminidase release: IC_50_ > 40 µM	*Cudrania tricuspidata*					AI			[62]
5′-geranyl-4′-methoxy-5,7,2′-trihydroxyflavone	AChE inhibition: IC_50_ = 3.1 µM BChE inhibition: IC_50_ = 1.74 µM	*Morus alba*							AA	[136,147,148]
5′-geranyl-5,7,2′,4′-tetrahydroxy-flavone	BChE inhibition: IC_50_ = 16.21 µM								AA	[136,147,148]
	MIC =2–4 μg mL^−1^ MBC = 2–8 μg mL^−1^				AM					[82]
5RE4-artocarpin	MBA = −6.6 kcal mol^−1^	*Artocarpus altilis*			AM					[67]
5RE4-artoindonesianin V	MBA = −6.6 kcal mol^−1^				AM					[67]
6-[(1R*,6R*)-3-methyl-6-(1-methylethenyl)-2-cyclohexen-1-yl]-5,7,4′-trihydroxyisoflavone	IC_50_ = 32.5 ± 6.7 µM	*Ficus tikoua*	AD							[29]
6-prenylapigenin	MIC = 0.8 μg mL^−1^	*Dorstenia barteri*			AM					[71]
6,8-diprenyleriodictyol	MIC = 0.5 μg mL^−1^				AM					[71]
7-O-(2,2-dimethylallyl)-aromadendrin	MIC = 32–128 μg mL^−1^	*Maclura pomifera*			AM					[75]
8-(1,1-dimethylallyl)-5′-(3-methyl-but-2-enyl)-3′,4′,5,7-tetrahydroxyflavonol	IC_50_ = 3.6 ± 0.4 µM	*Broussonetia papyrifera*	AD							[25]
8-Geranyl-3-(hydroxyprenyl) isoetin	Tyrosinase inhibition: IC_50_ > 300.0 µM	*Artocarpus altilis*						EI		[98]
8-Geranylapigenin	Tyrosinase inhibition: IC50 > 300.0 µM							EI		[98]
	HeLa: IC_50_ = 2.24 ± 0.48 µM MCF-7: IC_50_ = 3.21 ± 0.87 µM	*Morus alba*		AC						[56]
Albanin T	IC_50_ = 35.35 ± 0.42 µM		AD							[33]
Albanol B	AChE inhibition: IC_50_ = 2.47 ± 0.06 BChE inhibition: IC_50_ = 1.39 ± 0.06								AA	[136]
Alpinumisoflavone	Inhibition of β-hexosaminidase release: IC_50_ > 40 µM	*Cudrania tricuspidata*					AI			[62]
	MIC = 4–128 μg mL^−1^	*Maclura pomifera*			AM					[75]
Alpinumisoflavone, 6,8-diprenylgenistein	ND	*Maclura tricuspidata*	AD							[30]
Amentoflavone	MIC = 0.7 µg mL^−1^	*Dorstenia barteri*			AM					[71]
Angusticornin B	MIC = 78.12 µg mL^−1^	*Dorstenia angusticornis*			AM					[70]
Artelasticin	MCF-7: IC_50_ = 8.9 ± 2.4	*Artocarpus elasticus*		AC						[149]
	TK-10: IC_50_ = 10.6 ± 3.1 µM UACC-62: IC_50_ = 8.8 ± 0.2 µM			AC						
	MCF-7: IC_50_ = 2.2 ± 0.3 µM			AC						[43]
	MCF-7: IC_50_ = 2.2 ± 0.3 µM			AC						
	TK-10: IC_50_ = 4.6 ± 1.7 µM			AC						
	UACC-62: IC_50_ = 2.2 ± 0.5 µM			AC						
	NCI–H460: IC_50_ = 5.2 ± 1.5 µM			AC						
	SF-268: IC_50_ = 10.8 ± 1.8 µM			AC						
Artelastocarpin	MCF-7: IC_50_ = 7.1 ± 0.3 µM TK-10: IC_50_ = 12.5 ± 0.1 µM UACC-62: IC_50_ = 8.5 ± 0.3 µM			AC						[149]
Artelastochromene	MCF-7: IC_50_ = 9.6 ± 0.7 µM TK-10: IC_50_ = 10.5 ± 1.1 µM UACC-62: IC_50_ = 10.5 ± 1.7 µM			AC						[43]
Artelastoheterol	IC_50_ = 24.2 ± 0.8 µM					AO				[90]
Artocarpesin	MCF-7: IC_50_ = 42.4 ± 1.6 µM TK-10: IC_50_ = 64.1 ± 5.7 µM UACC-62: IC_50_ = 38.1 ± 2.4 µM			AC						[149]
Artocarpetin	ND	*Artocarpus heterophyllis*				AO				[84,85]
Artocarpetin A	ND					AO				
Artocarpin	MIC = 3.12 μg mL^−1^	*Artocarpus altilis*			AM					[98]
	MIC = 2 µg mL^−1^ MIC = 4 µg mL^−1^	*Artocarpus integer*			AM					[58]
	Tyrosinase inhibition: IC_50_ = 270.3 µM	*Artocarpus altilis*						EI		[98]
	ND	*Artocarpus heterophyllis*				AO				[84]
Artoflavone A	Tyrosinase inhibition: IC_50_ > 300.0 µM	*Artocarpus altilis*						EI		[98]
	ND	*Artocaspus communis*				AO				[91]
Artogomezianone	Tyrosinase inhibition: IC_50_ = 84.8 µM	*Artocarpus altilis*						EI		[98]
Artonin A	ND	*Artocarpus heterophyllis*				AO				[85]
Artonin B	ND					AO				
Artonin E	IC_50_ = 0.2 μg mL^−1^	*Artocarpus rigida*			AM					[66]
	P388: IC_50_ = 0.06 µM	*Artocarpus elasticus*		AC						[150]
	MDA-MB231: IC_50_ = 9.77 ± 0.50 µM			AC						[46]
	MCF-10A: IC_50_ = 45.80 ± 3.60 µM			AC						
	IC_50_ = 0.2 µg mL^−1^				AM					[66]
	IC_50_ = 0.1 µg mL^−1^	*Artocarpus heterophyllus*			AM					
	IC_50_ = 0.3 µg mL^−1^	*Artocarpus lanceifolius*			AM					
Atalantoflavone	HeLa: IC_50_ = 1.25 ± 0.46 µM MCF-7: IC_50_ = 6.54 ± 1.23 µM	*Morus alba*		AC			AI			[56]
Auriculatin	MIC = 1–8 μg mL^−1^	*Maclura pomifera*			AM					[75]
Bartericin	ND	*Dorstenia barteri*				AO				[70]
Bartericin A	MIC = 0.31 µg mL^−1^	*Dorstenia angusticornis*			AM					[70]
	MIC = 39.06 µg mL^−1^				AM					[70]
Broussochalcone A	IC_50_ = 5.3 ± 0.3 µM	*Broussonetia papyrifera*	AD							[25]
	MIC = 45 µg mL^−1^				AM					[25]
Broussochalcone B	IC_50_ = 11.1 ± 0.5 µM		AD							[25]
Broussonol A	A549: IC_50_ = 8.74 µM	*Broussonetia kazinoki*		AC						[45]
	HCT-8: IC_50_ = 9.10 µM			AC						[45]
	KB: IC_50_ > 10 µM			AC						[45]
Broussonol B	A549: IC_50_ = 5.52 µM			AC						[45]
	HCT-8: IC_50_ = 8.80 µM			AC						[45]
	KB: IC_50_ > 10 µM			AC						[45]
Broussonol C	A549: IC_50_ = 7.77 µM			AC						[45]
	HCT-8: IC_50_ = 9.63 µM			AC						[45]
	KB: IC_50_ > 10 µM			AC						[45]
Broussonol D	A549: IC50 > 10 µM						AI			[151]
	HCT-8: IC50 > 10 µM						AI			[151]
	KB: IC50 = 4.15 µM						AI			[151]
	A549: IC_50_ > 10 µM			AC						[45]
	HCT-8: IC_50_ > 10 µM			AC						[45]
	KB: IC_50_ = 4.15 µM			AC						[45]
Broussonol E	A549: IC_50_ > 10 µM			AC						[45]
	HCT-8: IC_50_ > 10 µM			AC						[45]
	KB: IC_50_ > 10 µM			AC						[45]
	A549: IC50 > 10 µM							EI		[151]
	HCT-8: IC50 > 10 µM							EI		[151]
	KB: IC50 > 10 µM							EI		[151]
Carpelastofuran	MCF-7: IC_50_ = 8.7 ± 0.5 µM	*Artocarpus elasticus*		AC						[149]
	TK-10: IC_50_ = 12.3 ± 1.9 µM UACC-62: IC_50_ = 8.9 ± 0.3 µM	*Artocarpus elasticus*		AC						[43]
Chalcomoracin	IC_50_ = 2.59 ± 0.24 µM	*Morus alba*	AD							[31]
Cochinchinone A	MIC= 32–128 μg mL^−1^	*Maclura pomifera*			AM					[75]
Corylifol C	IR = >65%	*Artocarpus altilis*			AM					[62]
Cudracusisoflavone M	ND	*Maclura tricuspidata*	AD							[30]
Cudracuspixanthone A	IC_50_ = 1.9 ± 0.4 µM	*Cudrania tricuspidata*	AD							[26]
Cudraflavanone D	IC_50_ = 5.7 ± 1.5 µM		AD							[26]
Cudraflavanone G	Against MPP+-induced cell death: EC_50_ > 50 µM								AA	[141]
	Against OGD-induced neurotoxicity: EC_50_ > 50 µM								AA	[141]
	Against 6-OHDA-induced cell death: EC_50_ > 50 µM								AA	[141]
Cudraflavone B	MIC = 12.5–50 μg mL^−1^	*Artocarpus altilis*			AM					[98]
	COX-1 inhibition: IC_50_ = 1.5 µM	*Morus alba*					AI			[152]
	COX-2 inhibition: IC_50_ = 2.5						AI			[109]
	IC_50_ = 20.7 ± 2.5 µM	*Morus nigra*	AD							[35]
Cudraflavone C	MIC = 12.5–50 μg mL^−1^	*Artocarpus altilis*			AM					[98]
	MIC = 4 μg mL^−1^	*Artocarpus hirsutus*			AM					[58]
Cudraflavone H	Against MPP+-induced cell death: EC_50_ > 50 µM	*Cudrania tricuspidata*							AA	[141]
	Against OGD-induced neurotoxicity: EC_50_ > 50 µM								AA	[141]
	Against 6-OHDA-induced cell death: EC_50_ > 50 µM								AA	[141]
Cudraxanthone L	IC_50_ = 4.6 ± 0.8 µM		AD							[26]
Cycloartelastoxanthone	IC_50_ = 18.7 ± 2.2 µM	*Artocarpus elasticus*				AO				[90]
	IC_50_ = 26.8 ± 1.2 µM					AO				
Cycloartocarpin	Tyrosinase inhibition: IC_50_ > 300.0 µM	*Artocarpus altilis*						EI		[98]
Cyclocommunin	MCF-7: IC_50_ = 21.8 ± 1.3 µM	*Artocarpus elasticus*		AC						[149]
	TK-10: IC_50_ = 40.5 ± 1.4 µM UACC-62: IC_50_ = 26.6 ± 3.2 µM			AC						[149]
Cyclocommunol	Tyrosinase inhibition: IC_50_ = 209.1 µM	*Artocarpus altilis*						EI		[98]
	P388: IC_50_ = 0.2 µM	*Artocarpus elasticus*		AC						[150]
	MIC = 8–16 μg mL^−1^	*Morus alba*			AM					[80]
Cyclogeracommunin	Tyrosinase inhibition: IC_50_ > 300.0 µM	*Artocarpus altilis*						EI		[98]
	IC_50_ of 73.3 ± 19.1 µM	*Artocarpus elasticus*				AO				[90]
Cycloheterophyllin	ND	*Artocarpus heterophyllis*				AO				[92]
	ND					AO				
Cyclomorusin	AChE, BChE, BACE1 inhibition: IC_50_ > 100 µM	*Morus alba*							AA	[136,147,148]
	IC_50_ = 38.81 ± 10.39 µM		AD							[34]
	Tyrosinase inhibition: IC_50_ > 300.0 µM	*Artocarpus altilis*						EI		[98]
	HeLa: IC_50_ = 1.66 ± 0.27 µM	*Morus alba*		AC						[56]
	MCF-7: IC_50_ = 7.85 ± 1.30			AC						[56]
Cyclomulberrin	HeLa: IC_50_ = 3.69 ± 0.86 µM			AC						[56]
	MCF-7: IC_50_ = 7.19 ± 0.77 µM			AC						[56]
	HeLa: IC_50_ = 3.69 ± 0.86 µM							EI		[56]
	MCF-7: IC_50_ = 7.19 ± 0.77 µM							EI		[56]
Derrone	Inhibition of β-hexosaminidase release: IC_50_ > 40 µM	*Cudrania tricuspidata*					AI			[62]
	MIC = 4–128 μg mL^−1^	*Maclura pomifera*			AM					[75]
Dorsilurin C	α-D-glucosidase inhibition: IC_50_ = 11.17 µM	*Dorstenia psilurus*	AD							[27]
	β-D-glucosidase inhibition: IC_50_ = 422.21 µM β-D-mannosidase inhibition: IC_50_ = 358.21 µM		AD							[27]
Dorsilurin F (6,8,4′-triprenyl-5,7,3′-trihydroxyflavonol)	α-D-glucosidase inhibition: IC_50_ = 4.13 ± 0.12 µM		AD							[27]
	β-D-glucosidase inhibition: IC50 = 117.33 ± 0.15 µM		AD							[27]
	β-D-mannosidase inhibition: IC_50_ = 192.09 ± 0.63 µM		AD							[27]
Dorsilurin G	α-D-glucosidase inhibition: IC_50_ = 7.51 µM		AD							[27]
	β-D-glucosidase inhibition: IC_50_ = 431.14 µM		AD							[27]
	β-D-mannosidase inhibition: IC_50_ = 231.99 µM		AD							[27]
Dorsilurin H	α-D-glucosidase inhibition: IC_50_ = 24.01 µM		AD							[27]
	β-D-glucosidase inhibition: IC_50_ = 671.03 µM		AD							[27]
Dorsilurin I	α-D-glucosidase inhibition: IC_50_ = 21.49 µM		AD							[27]
	β-D-glucosidase inhibition: IC_50_ = 431.14 µM		AD							[27]
Dorsilurin J	α-D-glucosidase inhibition: IC_50_ = 16.91 µM		AD							[27]
	β-D-glucosidase inhibition: IC_50_ = 316.55 µM		AD							[27]
	β-D-mannosidase inhibition: IC_50_ = 518.27 µM		AD							[27]
Dorsilurin K (5,6-7,8-bis(2,2-imethyldihydropyrano)-3′-hydroxy-4′-prenyl-flavonol)	α-D-glucosidase inhibition: IC_50_ = 43.95 ± 0.46 µM		AD							[27]
Elastichalcone B	MBC = 156.02	*Artocarpus elasticus*			AM					[57]
Elastichalcone C	SA: IC_50_ = 19.5 µM MRSA: IC_50_ = 9.75 µM				AM					[153]
Elastichalcone D	ND				AM					[153]
Elastichalcone E	ND				AM					[153]
Elastichalcone F	MRSA: MBC 174.79 µM				AM					[153]
Erysenegalensein E	Inhibition of β-hexosaminidase release: IC_50_ > 40 µM	*Cudrania tricuspidata*					AI			[62]
Erythrinin C	IC_50_ = 23.5 µM	*Maclura tricuspidata*	AD							[30]
Euchrenone b_10_	ND		AD							[30]
Ficucaricone D	Inhibition of NO production: IC_50_ = 2.06 µM	*Ficus carica*					AI			[28]
Ficusin A	ND	*Ficus hispida*	AD							[28]
	IC_50_ = 84.6 ± 7.8 µM	*Ficus tikoua*	AD							[29]
Furanocyclocommunin	Tyrosinase inhibition: IC_50_ > 220.0 µM	*Artocarpus altilis*						EI		[98]
Gancaonin A	Inhibition of β-hexosaminidase release: IC_50_ > 40 µM	*Cudrania tricuspidata*					AI			[62]
Gancaonin M	ND	*Maclura tricuspidata*	AD							[30]
	MIC = 2–8 μg mL^−1^	*Maclura cochinchinensis*			AM					[74]
Gancaonin Q	MIC = 0.61 µg mL^−1^	*Dorstenia angusticornis*			AM					[70]
Hesperetin	AChE, BChE, BACE1 inhibition: IC_50_ > 100 µM	*Morus alba*							AA	[136,147,148]
Heteroflavone C	IC_50_ = 6.3 nmol L^–1^	*Artocarpus champeden*			AM					[64]
Icariin	ND	*Morus alba*							AA	[136]
Isobavachalcone	MIC = 0.3 µg mL^−1^	*Dorstenia barteri*			AM					[71]
	MIC = 1–4 μg mL^−1^				AM					[72]
	MIC = 0.5 μg mL^−1^				AM					[72]
	ND	*Artocarpus anisophyllus*				AO				[87]
	ND	*Artocarpus scortechinii*				AO				[88]
Isocycloartobiloxanthone	Tyrosinase inhibition: IC_50_ = 279.5 µM	*Artocarpus altilis*						EI		[98]
Isoderrone	IC_50_ = 108.1 ± 10.8 µM	*Ficus hispida*	AD							[28]
Isoerysenegalensein E	Inhibition of β-hexosaminidase release: IC_50_ > 40 µM	*Cudrania tricuspidata*					AI			[62]
	ND	*Maclura tricuspidata*	AD							[30]
Kanzonol C	MIC = 4.9 µg mL^−1^	*Dorstenia barteri*			AM					[71]
Kazinol A	IC_50_ = 12.0 ± 0.8 µM	*Broussonetia papyrifera*	AD							[25]
Kazinol B	MIC = 20 µg mL^−1^				AM					[68]
Kazinol E	IC_50_ = 10.6 ± 1.5 µM		AD							[25]
Kuwanon A	AChE, BChE, BACE1 inhibition: IC_50_ > 100 µM	*Morus alba*							AA	[136,147,148]
Kuwanon B	MIC = 1.6 μmol L^−1^ µM				AM					[79]
Kuwanon C	ND	*Morus lhou*							AA	[137]
	MICs of 4–8 μg mL^−1^	*Morus alba*			AM					[77,78]
	MIC = 2 and 8 μg mL^−1^				AM					[81]
	Reducing the secretion of TNF-α: IC_50_ < 10 µM						AI			[52]
	MCF-7: IC_50_ = 3.92 µM	*Morus nigra*		AC						[154]
	HepG2: IC_50_ = 9.54 µM			AC						[154]
	MCF-7: IC_50_ = 3.92 µM						AI			[154]
	HepG2: IC_50_ = 9.54 µM						AI			[154]
	MCF-7: IC_50_ = 3.92 µM							EI		[154]
	HepG2: IC_50_ = 9.54 µM							EI		[154]
Kuwanon E	AChE, BChE, BACE1 inhibition: IC_50_ > 100 µM	*Morus alba*							AA	[136,147,148]
	AChE, BChE, BACE1 inhibition: IC_50_ > 100 µM								AA	[136,147,148]
	MICs of 2–8 μg mL^−1^				AM					[80]
	MIC = 2 and 8 μg mL^−1^				AM					[81]
	MIC = 4–16 μg mL^−1^				AM					[81]
	Reducing the secretion of TNF-α and IL-1β: IC_50_ < 100 µM						AI			[52]
Kuwanon G	ChEs inhibition: IC_50_ = 20.4–37.07 μM								AA	[136]
	MCF-7: IC_50_ = 34.35 µM	*Morus nigra*		AC						[154]
	HepG2: IC_50_ = 35.79 µM			AC						[154]
Kuwanon S	HeLa: IC_50_ = 1.64 ± 0.21 µM	*Morus alba*		AC						[56]
	MCF-7: IC_50_ = 7.02 ± 1.66 µM			AC						[56]
Kuwanon T	IC_50_ = 10.53 ± 1.10 µM		AD							[34]
	MICs of 2–8 μg mL^−1^				AM					[81]
	MIC = 2 and 8 μg mL^−1^				AM					[81]
Kuwanon U	AChE, BChE, BACE1 inhibition: IC_50_ > 100 µM								AA	[136,147,148]
	MIC = 4–16 μg mL^−1^				AM					[79]
	MBC = 8–32 μg mL^−1^				AM					[79]
	MIC = 2 and 8 μg mL^−1^				AM					[81]
Lupalbigenin	MIC = 1 μg mL^−1^	*Maclura cochinchinensis*			AM					[74]
Lupiwighteone	MIC = 2–8 μg mL^−1^				AM					[74]
	MIC = 128 μg mL^−1^				AM					[74]
Macluracochinone E	MIC = 1–8 μg mL^−1^				AM					[74]
Macluracochinone A	MIC= 2–8 μg mL^−1^				AM					[74]
Macluracochinone B	MIC= 2–8 μg mL^−1^				AM					[74]
Macluracochinone C	MIC= 1–8 μg mL^−1^				AM					[74]
Macluracochinone D	MIC= 1–8 μg mL^−1^				AM					[74]
Millewanin G	IC_50_ = 3.2 µM	*Maclura tricuspidata*	AD							[30]
Millexatin F	MIC = 2–8 μg mL^−1^	*Maclura pomifera*			AM					[75]
Moracin C	IC_50_ = 4.04 ± 0.84 µM	*Morus alba*	AD							[31]
Moracin N	IC_50_ = 2.76 ± 0.3 µM		AD							[31]
Mormin	AChE, BChE, BACE1 inhibition: IC_50_ > 100 µM								AA	[136,147,148]
Morunigrol B	IC_50_ = 7.7 ± 0.9 µM	*Morus nigra*	AD							[35]
Morunigrol A	IC_50_ = 12.5 ± 1.3 µM		AD							[35]
Morunigrol C	IC_50_ = 5.3 ± 1.8 µM		AD							[35]
Morusin	ND	*Morus alba*							AA	[136,147,148]
	ND	*Morus lhou*							AA	[137]
	IC_50_ = 22.1 ± 2.9 µM	*Morus nigra*	AD							[35]
	MIC = 12.5–50 μg mL^−1^	*Artocarpus altilis*			AM					[98]
	HeLa: IC_50_ = 0.64 ± 0.14	*Morus alba*		AC						[56]
	MCF-7: IC_50_ = 7.88 ± 1.89			AC						[56]
	MICs of 4–8 μg mL^−1^				AM					[81]
	MIC = 1 μg mL^−1^				AM					[82]
	IC_50_ = 1819.83 ± 144.53 µM					AO				[105]
	IC_50_ = 297.83 ± 7.27 µM					AO				
	HeLa: IC_50_ = 0.64 ± 0.14 µM						AI			[56]
	MCF-7: IC_50_ = 7.88 ± 1.89 µM						AI			[56]
	MIC = 8–32 μg mL^−1^				AM					[80]
	IC_50_ = 3.19 ± 2.10 µM		AD							[32]
Morusinol	AChE, BChE, BACE1 inhibition: IC_50_ > 100 µM								AA	[136,147,148]
	MIC = 2 and 8 μg mL^−1^				AM					[81]
	Reducing the secretion of TNF-α: IC_50_ < 10 µM						AI			[52]
Morusyunnansin C	Tyrosinase inhibition: IC_50_ > 25 µM	*Morus yunnanensis*						EI		[130]
Morusyunnansin D	Tyrosinase inhibition: IC_50_ > 25 µM							EI		[130]
Morusyunnansin E	Tyrosinase inhibition: IC_50_ = 1.43 µM							EI		[130]
Morusyunnansin F	Tyrosinase inhibition: IC_50_ = 0.15 µM							EI		[130]
Mulberrofuran G	AChE inhibiton: IC_50_ = 2.13 ± 0.02 µM	*Morus alba*							AA	[136]
	BACE1 inhibition: IC_50_ = 0.31 ± 0.01	*Morus alba*							AA	[136]
Myrsininone A	ND	*Ficus hispida*	AD							[28]
Neocyclomorusin	AChE, BChE, BACE1 inhibition: IC_50_ > 100 µM	*Morus alba*							AA	[136,147,148]
Nigrasin H	IC_50_ = >50 µM	*Morus nigra*	AD							[36]
Nigrasin I	IC_50_ = >25 µM	*Morus nigra*	AD							[36]
Papyriflavonol A	IC_50_ = 2.1 ± 0.2 µM	*Broussonetia papyrifera*	AD							[25]
	MIC = 10 MIC = 12.5 µg mL^−1^	*Broussnetia papyrifera*			AM					[68]
Sanggenol A	ND	*Morus alba*			AM					[75]
Sanggenon C	MIC = 70.6 μmol L^−1^				AM					[78,79]
Sanggenon D	MIC = 17.6 μmol L^−1^				AM					[76]
Sanggenon E	Reducing the secretion of TNF-α: IC_50_ < 10 µM						AI			[52]
Sanggenon H	Reducing the secretion of TNF-α: IC_50_ < 10 µM						AI			[52]
Sanggenon J	HeLa: IC_50_ = 2.28 ± 0.04 µM			AC						[56]
	MCF-7: IC_50_ = 4.56 ± 0.71 µM			AC						[56]
Sanggenon K	HeLa: IC_50_ = 2.29 ± 1.64 µM			AC						[56]
	MCF-7: IC_50_ = 3.51 ± 0.59 µM			AC						[56]
Senegalensin	ND	*Maclura tricuspidata*	AD							[30]
Soroceal	Reducing the secretion of TNF-α: IC_50_ < 10 µM	*Morus alba*					AI			[52]
Stipulin	MIC = 78.12 µg mL^−1^	*Dorstenia angusticornis*			AM					[70]
Tomentodiplacone L	Reducing the secretion of TNF-α: IC_50_ = 6.7 µM	*Morus alba*					AI			[52]
Warangalone (scandenone)	Inhibition of β-hexosaminidase release: IC_50_ > 40 µM	*Cudrania tricuspidata*					AI			[62]
	MIC = 1–8 μg mL^−1^	*Maclura cochinchinensis*			AM					[74]
	MIC = 4 μg mL^−1^	*Maclura pomifera*			AM					[75]
Xanthone V1	MIC = 32–128 μg mL^−1^	*Maclura pomifera*			AM					[75]
Xanthone-like compound	IC_50_ = 42.2 ± 2.8	*Artocarpus elasticus*				AO				[86]

AD (anti-diabetic); AC (anti-cancer); AM (antimicrobial); AO (antioxidant); AI (anti-inflammatory); EI (enzymatic inhibition); AA (anti-Alzheimer); ND (not determined); MBC (minimum bactericidal concentration); MIC (minimum inhibitory concentration); EC_50_ (median effective concentration).
